# The proteome and phosphoproteome of circulating extracellular vesicle-enriched preparations are associated with characteristic clinical features in type 1 diabetes

**DOI:** 10.3389/fendo.2023.1219293

**Published:** 2023-07-28

**Authors:** Anna Casu, Yury O. Nunez Lopez, Gongxin Yu, Christopher Clifford, Anika Bilal, Alejandra M. Petrilli, Heather Cornnell, Elvis Alvarez Carnero, Ananya Bhatheja, Karen D. Corbin, Anton Iliuk, David M. Maahs, Richard E. Pratley

**Affiliations:** ^1^ AdventHealth, Translational Research Institute (TRI), Orlando, FL, United States; ^2^ Biomarker Discovery Department, Tymora Analytical Operations, West Lafayette, IN, United States; ^3^ Department of Pediatrics, Stanford University School of Medicine, Stanford, CA, United States

**Keywords:** extracellular vesicles, proteomics, phosphoproteomics, type 1 diabetes, human

## Abstract

**Introduction:**

There are no validated clinical or laboratory biomarkers to identify and differentiate endotypes of type 1 diabetes (T1D) or the risk of progression to chronic complications. Extracellular vesicles (EVs) have been studied as biomarkers in several different disease states but have not been well studied in T1D.

**Methods:**

As the initial step towards circulating biomarker identification in T1D, this pilot study aimed to provide an initial characterization of the proteomic and phosphoproteomic landscape of circulating EV-enriched preparations in participants with established T1D (N=10) and healthy normal volunteers (Controls) (N=7) (NCT03379792) carefully matched by age, race/ethnicity, sex, and BMI. EV-enriched preparations were obtained using EVtrap^®^ technology. Proteins were identified and quantified by LC-MS analysis. Differential abundance and coexpression network (WGCNA), and pathway enrichment analyses were implemented.

**Results:**

The detected proteins and phosphoproteins were enriched (75%) in exosomal proteins cataloged in the ExoCarta database. A total of 181 proteins and 8 phosphoproteins were differentially abundant in participants with T1D compared to controls, including some well-known EVproteins (i.e., CD63, RAB14, BSG, LAMP2, and EZR). Enrichment analyses of differentially abundant proteins and phosphoproteins of EV-enriched preparations identified associations with neutrophil, platelet, and immune response functions, as well as prion protein aggregation. Downregulated proteins were involved in MHC class II signaling and the regulation of monocyte differentiation. Potential key roles in T1D for C1q, plasminogen, IL6ST, CD40, HLA-DQB1, HLA-DRB1, CD74, NUCB1, and SAP, are highlighted. Remarkably, WGCNA uncovered two protein modules significantly associated with pancreas size, which may be implicated in the pathogenesis of T1D. Similarly, these modules showed significant enrichment for membrane compartments, processes associated with inflammation and the immune response, and regulation of viral processes, among others.

**Discussion:**

This study demonstrates the potential of proteomic and phosphoproteomic signatures of EV-enriched preparations to provide insight into the pathobiology of T1D. The WGCNA analysis could be a powerful tool to discriminate signatures associated with different pathobiological components of the disease.

## 1 Introduction

Type 1 diabetes (T1D) is a chronic disease characterized by hyperglycemia due to the loss of β cells and the consequent lack of endogenous insulin secretion. The loss of β-cell function and mass is due to a targeted autoimmune process directed against these cells, but the precise causes are still largely unknown. Although classically described as a disease of childhood, more than half of all incident cases of T1D now occur in adults, who tend to have a slower evolution of the disease. At present, there are no validated clinical or laboratory biomarkers able to predict the progression rate from antibody positivity to clinically manifest disease and to discriminate the different endotypes. Predictors of the rate of insulin secretion decline either before or after the clinical onset of the disease are also lacking (1–4). In addition to the loss of β-cell function, T1D also affects the exocrine pancreas. Pancreas size is 20–50% smaller in patients with T1D, and it is also smaller among individuals with multiple islet-related autoantibodies and first-degree relatives of patients with T1D (5). The mechanisms for this are not known, but these findings suggest that pancreas volume/mass could be a biomarker of T1D pathobiology (1). The recent approval of the first T1D disease-modifying therapy able to delay the onset of clinical diabetes in those at high risk highlights the need for additional novel biomarkers that could discriminate those who would most benefit from this therapy and those who would not (6) as well as provide therapeutic monitoring capacity (4).

Once established, T1D is treated with subcutaneous insulin administration to achieve blood and tissue glucose levels sufficiently close to the normal range to prevent or limit the development of micro and macrovascular chronic complications of the disease (7). However, there is a heterogeneity in the progression to chronic complications that is not completely explained by glycemic control. This is particularly true for cardiovascular disease (8) which is responsible for approximately two-thirds of deaths among adults with T1D. Additional biomarkers are therefore needed to improve the diagnostic capacity, predict progression and risk of chronic complications, and evaluate treatment efficacy.

Recent studies have highlighted the potential utility of circulating extracellular vesicles (EVs) as biomarkers in various disease states, including cancer, cardiovascular disease and diabetes (9, 10). EVs in their different forms (i.e., exosomes and microvesicles) originate from virtually all tissues, are secreted into the interstitial space, and can be found in circulation as well as in most bodily fluids. They play a key role in intercellular signaling as well as organ cross-talk. In diabetes, EVs may reflect the activation and regulation of physiologic and pathologic responses to β-cell stress or the autoimmune process. Consequently, they have been explored as biomarkers of metabolic derangement, insulin resistance, and chronic complications (11, 12). EV isolation could also increase the plasma proteome depth and could facilitate the discovery of relevant circulating plasma proteins as proposed in NOD mice (13).

Biomarker development requires a complex process that includes discovery, validation, clinical assay development and qualification as diagnostic tools (14). The discovery starts with the analysis of a small number of samples in well-phenotyped individuals, like the work described here. The use of EVs as biomarkers in T1D requires a proper understanding of the effect of different clinical components of the disease on the characteristics of circulating putative biomarkers. The EV proteome and phosphoproteome are of particular interest because of the possibility of identifying disease-specific signatures that, even if not directly pathogenetic, may be used as predictors of specific endotypes or of disease progression. However, there is a need to disentangle the modifications of circulating EV phenotype due to the metabolic derangement of the disease from those due to the pathogenic mechanisms per se. This pilot study explores the feasibility of using circulating EV-enriched preparations for this purpose in T1D.

In this cross-sectional exploratory analysis, we compared the proteome and phosphoproteome of EV-enriched preparations obtained from the serum of individuals with T1D and matched healthy controls who were deeply metabolically phenotyped. In addition, to discriminate proteomic signatures associated with either pathogenesis-specific or hyperglycemia-related clinical characteristics, we implemented an approach based on weighted gene coexpression network analysis (WGCNA) of the EV proteome and an extensive list of clinical metabolic features.

## 2 Materials and methods

### 2.1 Study population

The study (NCT03379792) was conducted according to the principles of the Declaration of Helsinki and followed GCP guidelines. All procedures were approved by the AdventHealth Institutional Review Board. Informed consent was obtained from all volunteers before initiation of the study. The goal of this pilot study was to characterize EV-enriched preparations in subjects with established T1D and carefully matched healthy controls. EVs were measured in archived serum samples from 17 subjects (N=10 with T1D and N=7 healthy volunteers without diabetes) and correlated with the clinical characteristics of the participants. The groups were specifically selected to be well balanced for most clinical characteristics and based on the availability of sufficient sample volume. This selection was automated using a custom script based on the *MatchIt* package (15) in the R programming environment.

### 2.2 In-depth clinical and metabolic phenotype

Anthropometric measures were performed according to standardized protocols. Blood samples were obtained in the morning after at least 10-hours of fasting. HbA1c levels were measured using a Cobas Integra 800 Analyzer (Roche, Basel, Switzerland). Body composition was measured using a GE Lunar iDEXA whole-body scanner (GE, Madison, WI). Whole body magnetic resonance imaging (MRI) was implemented using a Philips 3T Achieva MRI instrument (Philips Medical Systems MR Inc., Latham, NY). Briefly, participants were placed supine on the scanner and T1 and T2 weighted anatomical images were taken from their head through their toes. Participants were repositioned with a TorsoXL coil on their abdomen, to obtain higher resolution T1 and T2 images, and proton density fat fraction images. Images were analyzed on the Philips console and by manual and semiautomated segmentation using Analyze Pro (AnalyzeDirect Inc. Overland Park, KS, USA). Measurement of 24-hour energy expenditure and substrate oxidation rates was performed in whole room calorimeters at the AdventHealth Translational Research Institute according to standard methodology (16). Continuous glucose monitoring (CGM) (Dexcom G4, Dexcom Inc., San Diego, CA, USA) was used to monitor glucose dynamics over 10 days (17). A list of 155 clinical and EV related variables included in the study is presented in **Table 1** and **Supplementary Table ST1**.

**Table 1:** Relevant clinical characteristics of the study cohort.

Variable	Subgroup	Controls	T1D	p-value
**n**		7	10	
**Race [n (%)]**	Asian	1 (14.3)	0 (0.0)	0.245^§^
	Black	2 (28.6)	1 (10.0)	
	White	4 (57.1)	9 (90.0)	
**EthnicGroup [n (%)]**	Hispanic	4 (57.1)	4 (40.0)	0.279^§^
	Non-Hispanic	2 (28.6)	6 (60.0)	
	UNK	1 (14.3)	0 (0.0)	
**Sex (% F)**		71.4	60.0	1^§^
**Age (median [IQR])**		23.30 [23.00, 26.05]	25.35 [24.50, 28.23]	0.328^ǂ^
**BMI (median [IQR])**		28.90 [21.80, 30.45]	27.20 [24.12, 27.80]	0.769^ǂ^
**HbA1c (median [IQR])**		5.10 [4.95, 5.30]	8.10 [6.95, 9.55]	0.001^ǂ^
**Daily units of insulin/kg (median [IQR])**		NA	0.80 [0.56-0.95]	NA

^§^Fisher Exact Test. ^ǂ^Mann-Whitney U Test. T1D, Type 1 Diabetes; UKN, unknown; BMI, body mass index; HbA1c, Glycated hemoglobin; NA, not applicable.

### 2.3 EV isolation

Blood samples were allowed to coagulate at room temperature for 30 minutes after collection, then centrifuged at 1500 ×g for 15 min at 4°C to produce serum. Serum was stored at -80 °C until use. Frozen serum samples were thawed, then spun at 2,500 × g for 10 minutes. The pre-cleared samples were then diluted 20-fold in EVtrap dilution buffer and incubated with EVtrap beads for 30 min (18). After supernatant removal using a magnetic separator rack, the beads were washed with PBS, and the bonded fractions were eluted by a 10 min incubation with 200 mM triethylamine (TEA, Millipore-Sigma) and the resulting EV-enriched samples fully dried in a vacuum centrifuge.

### 2.4 Nanoparticle tracking analysis

The size distribution and concentration of particles in EV-enriched preparations were analyzed using dynamic light-scattering technology with a NanoSight NS300 instrument and NTA-3.4 software (Malvern Panalytical, Malvern). The instrument was equipped with a 488 nm blue laser module, flow-cell top plate, integrated temperature control, and a single-syringe pump module. Samples were diluted using cell culture grade water (Corning cat# 25-005-CI) to produce an optimal particle concentration for final measurement in the range of 10^7^ to 10^9^ particles/ml. Final quantification included 5 standard measurements of 1 minute of duration each, taken at a controlled temperature of 25°C and under constant automatic flow. Camera level for video capture was set to 12 and detection threshold to 5 for all sample measurements.

### 2.5 Mass spectrometry (LC-MS/MS)-based methods used to detect the global proteome and phosphoproteome of EV-enriched preparations

The isolated and dried EV-enriched samples were processed as described previously (18). Briefly, EV-enriched samples were lysed to extract proteins using the phase-transfer surfactant (PTS) aided procedure, normalized based on protein concentration using BCA assay, and the proteins digested with Lys-C (Fujifilm Wako Chemicals, Richmond, VA, USA) at 1:100 (wt/wt) enzyme-to-protein ratio for 3 h at 37°C. Trypsin was added to a final 1:50 (wt/wt) enzyme-to-protein ratio for overnight digestion at 37°C. After surfactant removal, the resulting peptides were desalted using Top-Tip C18 tips (Glygen Corp., Columbia, MD, USA) according to manufacturer’s instructions. Each sample was split into 99% and 1% aliquots for phosphoproteomic and proteomic experiments respectively. The samples were dried completely in a vacuum centrifuge and stored at -80°C. For phosphoproteome analysis, the larger portion of each sample was subjected to phosphopeptide enrichment using the PolyMAC Phosphopeptide Enrichment kit (Tymora Analytical, West Lafayette, IN, USA) according to manufacturer’s instructions, and the eluted phosphopeptides dried completely in a vacuum centrifuge. For phosphoproteomic analysis the whole enriched sample was used, while for proteomics only 50% of the sample was loaded onto the LC-MS.

Each dried peptide or phosphopeptide sample was dissolved at 0.1 μg/μL in 0.05% trifluoroacetic acid with 3% (vol/vol) acetonitrile. 10 μL of each sample was injected into an Ultimate 3000 nano UHPLC system (Thermo Fisher Scientific, Waltham, MA). Peptides were captured on a 2-cm Acclaim PepMap trap column (Thermo Fisher Scientific) and separated on a heated 50-cm column packed with ReproSil Saphir 1.9 μm C18 beads (Dr. Maisch GmbH, Ammerbuch-Entringen, Germany). The mobile phase buffer consisted of 0.1% formic acid in ultrapure water (buffer A) with an eluting buffer of 0.1% formic acid in 80% (vol/vol) acetonitrile (buffer B) run with a linear 60-min gradient of 6–30% buffer B at flow rate of 300 nL/min. The UHPLC was coupled online with a Q-Exactive HF-X mass spectrometer (Thermo Fisher Scientific). The mass spectrometer was operated in the data-dependent mode, in which a full-scan MS (from m/z 375 to 1,500 with the resolution of 60,000) was followed by MS/MS of the 15 most intense ions (30,000 resolution; normalized collision energy - 28%; automatic gain control target (AGC) - 2E4, maximum injection time - 200 ms; 60sec exclusion).

### 2.6 Bioinformatic analysis

The raw files were searched directly against the human Uniprot database with no redundant entries, using Byonic (Protein Metrics, Cupertino, CA) and Sequest search engines loaded into Proteome Discoverer 2.3 software (Thermo Fisher Scientific). MS1 precursor mass tolerance was set at 10 ppm, and MS2 tolerance was set at 20ppm. Search criteria included a static carbamidomethylation of cysteines (+57.0214 Da), and variable modifications of oxidation (+15.9949 Da) on methionine residues, acetylation (+42.011 Da) at N terminus of proteins, and phosphorylation of S, T and Y residues (+79.996 Da) for the phosphoproteomics data. Search was performed with full trypsin/P digestion and allowed a maximum of two missed cleavages on the peptides analyzed from the sequence database. The false-discovery rates of proteins and peptides were set at 0.01. All protein and peptide identifications were grouped and any redundant entries were removed. Only unique peptides and unique master proteins were reported.

All data were quantified using the label-free quantitation node of Precursor Ions Quantifier through the Proteome Discoverer v2.3 (Thermo Fisher Scientific). For the quantification of proteomic or phosphoproteomic data, the intensities of peptides/phosphopeptides were extracted with initial precursor mass tolerance set at 10 ppm, minimum number of isotope peaks as 2, maximum ΔRT of isotope pattern multiplets – 0.2 min, PSM confidence FDR of 0.01, with hypothesis test of ANOVA, maximum RT shift of 5 min, pairwise ratio-based ratio calculation, and 100 as the maximum allowed fold change. The abundance levels of all peptides and proteins were normalized using the total peptide amount normalization node in the Proteome Discoverer. For calculations of fold-change between the groups of proteins, total protein abundance values were added together and the ratios of these sums were used to compare proteins within different samples.

The proteomic and phosphoproteomic expression profiles of EV-enriched preparations were analyzed with a user-defined bioinformatic procedure that included raw data preprocessing, differential abundance analysis, weighted-gene correlation network analysis (WGCNA) (19), and enriched functional analyses. A Pearson correlation matrix of the proteins is used to form a hierarchical dendrogram that is then cut into branches corresponding to modules. Each module includes genes with similar expression pattern and most likely specific biological functions. The module eigengenes are also correlated with clinical traits and EV characteristics, generating a denser mechanistic overview of co-regulated proteins associated with the underlying T1D biology.

Briefly, missing values in the expression profiles were imputed, and the data were then log2-transformed, and scale-standardized. The imputation was performed with randomforest, an advanced machine-learning algorithm (20, 21). The normalized data were then analyzed using pvca (22) and limma (23), two R/Bioconductor software packages for biomarker discovery. Specifically, pvca, a package for principal variance component analysis, was first applied to identify significant covariates by fitting all “sources” as random effects, then linear models were created incorporating sex as covariate. Once established, the linear models were fitted using weighted least squares for each protein, moderated t-statistics, moderated F-statistics, log-fold changes and p-values of differential expression were calculated by empirical Bayes moderation of the standard errors.

The network module analysis was performed with WGCNA, an R package for weighted gene correlation network analysis (19). In short, it first performs a weighted protein co-expression network analysis to find clusters of highly correlated proteins (modules), and then relates modules to the clinical measurements and also with EV characteristics. Subsequent module membership, protein-trait significance and intra-module connectivity analysis was applied to identify the key driver proteins in modules of interest. KEGG pathway and Gene Ontology enrichment analyses were performed on the sets of differentially abundant proteins and phosphoproteins with clusterProfiler, an R package for enriched function analysis (24). Given a set of highly significant proteins identified, clusterProfiler suggests KEGG pathways and Gene Ontology (GO) functional groups significantly affected.

### 2.7 Statistical analysis

Differences in baseline clinical characteristics were assessed using the Mann–Whitney U test (for continuous variables) or the Fisher exact test (for categorical variables). For assessment of differential abundance of proteins and phosphoproteins of EV-enriched fractions, linear models using the *limma* package were implemented. Phosphoproteins that were also identified at the protein level, were subjected to differential abundance analysis of the phosphoprotein to protein ratios. Linear models included sex as covariate. Given the exploratory nature of our analysis, calculated effects and correlations with two-tailed P ≤ 0.01 and FDR<0.1 were considered significant. False discovery rates (FDR) correcting for multiple testing were calculated using the Benjamini-Hochberg correction as implemented for the *p.adjust* function in the *stats* package.

Being exploratory in nature, the study was not *a priori* powered to a specific objective. However, *a posteriori*, we calculated the statistical power to detect significantly differentially abundant proteins and phosphoproteins. For this, we determined the minimal, mean, and maximal standardize effect size that reached statistical significance with P<0.05 and FDR<0.1 (nominal P=0.0085 for proteomics and nominal P = 0.0009 for phosphoproteomics) and calculated the respective powers for linear models including a main effect variable (disease group) with 2 levels (NGT and T1D) and a cofactor variable (sex) also with 2 levels (Female and Male), using the *pwr* R package and the function *pwr.f2.test*. The nominal P values were defined *a posteriori* of the differential abundance analyses for proteins and phosphoproteins and represent the largest P values among the significantly (P<0.01 and FDR<0.1) differentially abundant features.

## 3 Results

### 3.1 Study design and clinical characteristics of the study cohort

The average age of the participants was 25.7 years (range 20.1-34.5), 11/17 were female, and the average BMI was 26.8 kg/m^2^ (range 19.5-38.6) for the whole population. The subset was not confounded by differences in variables known to affect metabolic function (e.g., age, BMI) as shown in **Table 1**. The comparison of all 155 demographic, anthropometric, metabolic, clinical, and EV-related characteristics for the study cohort are presented in **Supplementary Table ST1**. None of the participants with T1D had a history of chronic complications at the time of enrollment. Compared to healthy participants, participants with T1D had higher plasma glucose, HbA1c, time above 180 mg/dl and glucose variability obtained with CGM, larger kidneys and spleen when the volume was adjusted for weight, BMI or fat free mass compared to controls. In addition, participants with T1D had lower percentage of time between 69 and 54 mg/dl and time in range 70-180 mg/dl on CGM, calculated insulin sensitivity index according to the CACTI study, and smaller pancreas size compared to healthy participants, as expected.

### 3.2 EV-enriched preparations demonstrate enrichment in exosomal particles

**Figure 1:**
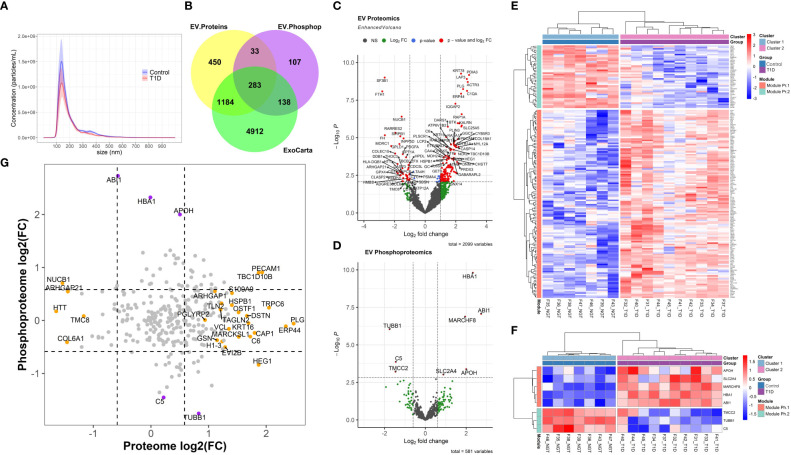
Extracellular Vesicle Characterization. **(A)** Nanoparticle Tracking Analysis (NTA) of extracellular vesicles (EVs) isolated from participants with type 1 diabetes (T1D) and Healthy normal volunteers (Controls). **(B)** Overlap between the exosomal proteins reported in Exocarta and all the EV proteins and phosphoproteins identified in the present work. **(C, D)** Volcano plots of the EV proteome and phosphoproteome. **(E, F)** Unsupervised clustering of the differentially abundant proteome and phosphoproteome, respectively. **(G)** Correlation plot of EV proteome versus phosphoproteome (grey circles: non-significant change, orange circles: differentially abundant only in proteomic analysis, purple circles: differentially abundant only in phosphoproteomic analysis; dark red circle: differentially abundant in both proteomic and phosphoproteomic analyses).

Circulating particles with a distribution of sizes compatible with small EVs/exosomes (**Figure 1A**) were isolated. The corresponding average and modal particle size was comparable between the groups (mean size 201.95 nm and modal size 143.8 nm in T1D and mean size 212.1 nm and modal size 145 nm in controls, p > 0.05, **Supplementary Table ST1**). Similarly, the total particle concentration did not differ between the two groups (0.82x10^11^ particles/ml in T1D and 1.25x10^11^ particles/mL in controls, p = 0.2, **Supplementary Table ST1**, **Figure 1A**). LC-MS/MS of EV-enriched preparations identified 1950 proteins, which included 1467 (75.2%) proteins reported in ExoCarta [a database which reports proteins, RNA, and lipids detected in exosomal preparations (25)], and more specifically, 91 of the top 100 proteins reported in this database (**Figure 1B**). Similarly, LC-MS/MS identified 561 phosphoproteins in circulating EV-enriched preparations, which included 421 (75%) of ExoCarta proteins (**Figure 1B**). These results support the conclusion that the isolated proteome and phosphoproteome are enriched for small EVs (exosomes).

### 3.3 The serum proteome and phosphoproteome of the EV-enriched preparations are different in people with T1D compared to controls

To characterize the circulating EV-enriched cargos in our study population, we conducted additional bioinformatic analyses including differential abundance analysis and unsupervised clustering analysis.

**Figure 2:**
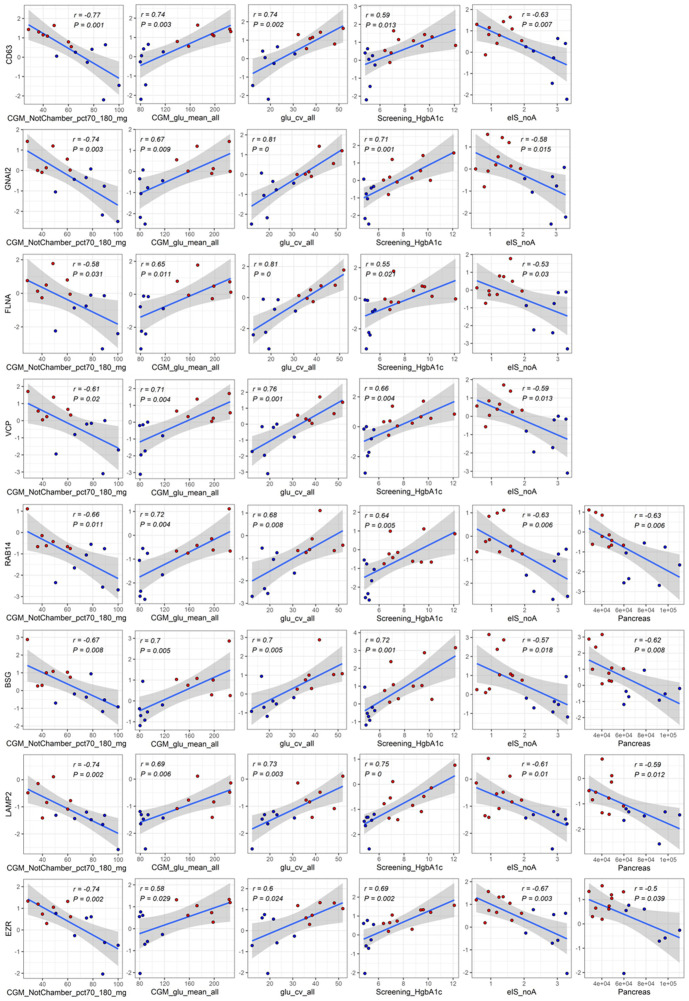
Significant correlations among differentially abundant top proteins in EV-enriched preparations and measures of glucose control, insulin, sensitivity, and pancreas size. Red: participants with type 1 diabetes (T1D). Blue: participants without diabetes (Controls). Abbreviations: CGM_NotChamber_pct70_180_mg: percentage of CGM reading between 70 and 180 mg/dl before admission to the metabolic chamber; CGM_glu_mean_all: average glucose obtained from CGM before and while in the metabolic chamber; glu_cv_all: coefficient of variation of all glucose measurements obtained with CGM; Screening_HgbA1c: glycated hemoglobin at screening visit; eIS_noA: estimated insulin sensitivity with a score not including adiponectin; Pancreas: pancreas size.

A total of 181 differentially abundant EV proteins were identified with p-value < 0.05 and FDR<0.1 between people with T1D and controls without diabetes. The range of absolute fold changes at this significance level was 1.64 to 7.29. Of these differentially abundant proteins, 135 were upregulated and 46 were downregulated in T1D, as compared to controls (**Table 2** and **Supplementary Table (ST)2**, **Figures 1C, E**). Eight EV-enriched phosphoproteins were different between the groups at the same level of significance (p-value ≤ 0.01 and FDR<0.1). The range of absolute fold changes was 1.84 to 6.63. Five of the differentially abundant phosphoproteins were upregulated and 3 were downregulated (**Table 3**, **Figures 1D, F**, phosphopeptides are reported in **ST3**). Notably, of the top 100 exosomal proteins reported by ExoCarta (25), 8 (CD63, RAB14, VCP, BSG (a.k.a. CD147), FLNA, GNAI2, LAMP2, EZR) were significantly upregulated at the protein level in the T1D group. These 8 top EV proteins correlated with clinical measures of glycemic control, insulin sensitivity, and four of them (i.e., RAB14, BSG, LAMP2, and EZR), also correlated with pancreas size (**Figure 2**). Pancreatic β-cell specific proteins were not among the differentially abundant proteins and phosphoproteins obtained from serum EV-enriched preparations probably because of the low abundance in circulation.

**Table 2:** Top 30 differentially abundant proteins in circulating EV-enriched preparations (T1D versus Controls).

Uniprot ID	Gene Symbol	Log2FC	log2 Ave Expr	P.Value	FDR
Q8N1N4	KRT78	2.34	0.83	5.49E-10	6.53E-07
P30101	PDIA3	2.87	0.5	6.75E-10	6.53E-07
O75533	SF3B1	-2.64	0.52	1.01E-09	6.53E-07
P28838	LAP3	2.72	0.13	1.24E-09	6.53E-07
P61158	ACTR3	2.78	0.25	1.96E-09	8.23E-07
P00747	PLG	2.48	0.32	5.86E-09	2.05E-06
P02745	C1QA	2.73	-0.03	7.70E-09	2.19E-06
P02794	FTH1	-2.8	-0.21	8.33E-09	2.19E-06
Q9BS26	ERP44	2.34	0.66	1.18E-08	2.75E-06
Q13576	IQGAP2	1.98	-0.81	5.52E-08	0.00001
P62834	RAP1A	2.38	-0.05	2.72E-07	5.20E-05
Q02818	NUCB1	-1.54	-1.02	3.93E-07	0.00007
Q06187	BTK	2.13	0.49	1.08E-06	0.00017
A0A0B4J1Y8	IGLV9-49	2.23	-0.19	1.12E-06	0.00017
O60229	KALRN	2.39	-0.51	1.55E-06	0.00022
P05141	SLC25A5	2.36	-0.18	1.80E-06	0.00023
P14868	DARS1	1.48	0.45	1.85E-06	0.00023
Q99969	RARRES2	-1.95	-0.42	4.67E-06	0.00054
P07954	FH	-2.63	-0.26	6.79E-06	0.00075
O00241	SIRPB1	-1.62	-0.72	7.94E-06	0.00083
P21281	ATP6V1B2	1.37	0.21	9.11E-06	0.00091
Q92835	INPP5D	-1.41	-1.09	1.12E-05	0.00107
O75223	GGCT	2.13	0.37	1.26E-05	0.00115
P16284	PECAM1	1.93	-0.17	1.63E-05	0.00142
O60664	PLIN3	1.88	0.78	2.06E-05	0.00173
P20936	RASA1	1.86	0.67	2.54E-05	0.00204
P80108	GPLD1	-1.68	0.35	2.62E-05	0.00204
P06576	ATP5F1B	1.68	-0.76	2.72E-05	0.00204
P08779	KRT16	1.69	0.27	2.82E-05	0.00204
P00387	CYB5R3	1.89	-0.81	3.12E-05	0.00218

Log2FC, base 2 logarithm of fold change; Log2 Ave Expr, base 2 logarithm of average expression; FDR, false discovery rate.

**Table 3:** Differentially abundant phosphoproteins in circulating EV-enriched preparations (T1D versus Controls).

Uniprot ID	Gene Symbol	Log2FC	log2 Ave Expr	p-value	FDR
Q8IZP0	ABI1	2.73	0.26	8.63E-08	2.51E-05
P69905	HBA1	2.32	0.57	1.56E-10	9.09E-08
P02749	APOH	2.00	0.20	4.13E-04	0.0399
Q5T0T0	MARCHF8	1.95	-0.52	1.31E-07	2.53E-05
P14672	SLC2A4	0.88	-0.95	9.48E-04	0.0689
P01031	C5	-1.45	-0.69	1.34E-04	0.0156
O75069	TMCC2	-1.47	-0.76	6.08E-04	0.0505
Q9H4B7	TUBB1	-1.76	0.06	8.95E-07	0.0001

Log2FC, base 2 logarithm of fold change; Log2 Ave Expr, base 2 logarithm of average expression; FDR, False discovery rate.

Our aim with this pilot study was to measure the degree of variability in circulating EV-enriched protein abundance and to assess our ability to identify associations to clinical characteristics and potentially expression of causative mechanisms (for example immune activation). The study was not *a priori* sized for a specific statistical power level. As shown in **Table 4**, the *a posteriori* power calculation demonstrated that we detected proteins with a mean standardize effect size (f2 = 1.507) with over 96% power, while proteins with a minimal significant differential abundance (minimal significant standardized effect size f2 = 0.712) were detected with approximately 67% power. Similarly, phosphoproteins with a mean standardize effect size (f2 = 1.721) were confidently detected with over 86% power, while phosphoproteins with a minimal significant differential abundance (minimal significant standardized effect size f2 = 0.88) were detected with approximately 45% power. These results will allow to estimate effective sample sizes for future validation studies using independent cohorts.

**Table 4:** Power calculation for 3 reference levels of significant differential abundance in proteins and phosphoproteins in circulating EV-enriched preparations (T1D versus Controls) (n=17).

Experiment	Effect Size category	Effect Size	Power (%)	Sig. Level
proteomics	Effect_Size_min	0.712	67.1	0.0085
proteomics	Effect_Size_mean	1.507	96.6	0.0085
proteomics	Effect_Size_max	2.867	100	0.0085
phosphoproteomics	Effect_Size_min	0.88	45.1	0.0009
phosphoproteomics	Effect_Size_mean	1.721	86.5	0.0009
phosphoproteomics	Effect_Size_max	2.729	98.6	0.0009

Since protein phosphorylation could provide information on cellular pathway regulation as described in cancer, we further dissected the changes in EV protein phosphorylation and assessed the relationship (correlation and comparison of phosphoprotein/protein ratios) between phosphoprotein and respective total protein levels, for those phosphoproteins that were also detected in the proteomic profiling experiment (a total of 288 phosphoproteins). As shown in **Figure 1G**, there was no significant correlation between the detected EV protein and EV phosphoprotein levels (r=-0.02, p=0.68). The differentially abundant features significantly changed in either the proteomic or the phosphoproteomic state, but not in both. In contrast, the comparison of phosphoprotein/protein ratios among the study groups identified nucleobindin-1 (NUCB1) with a highly significantly reduced ratio (logFC = -5.22, p = 2.84E-06, FDR = 0.00079).

**Figure 3:**
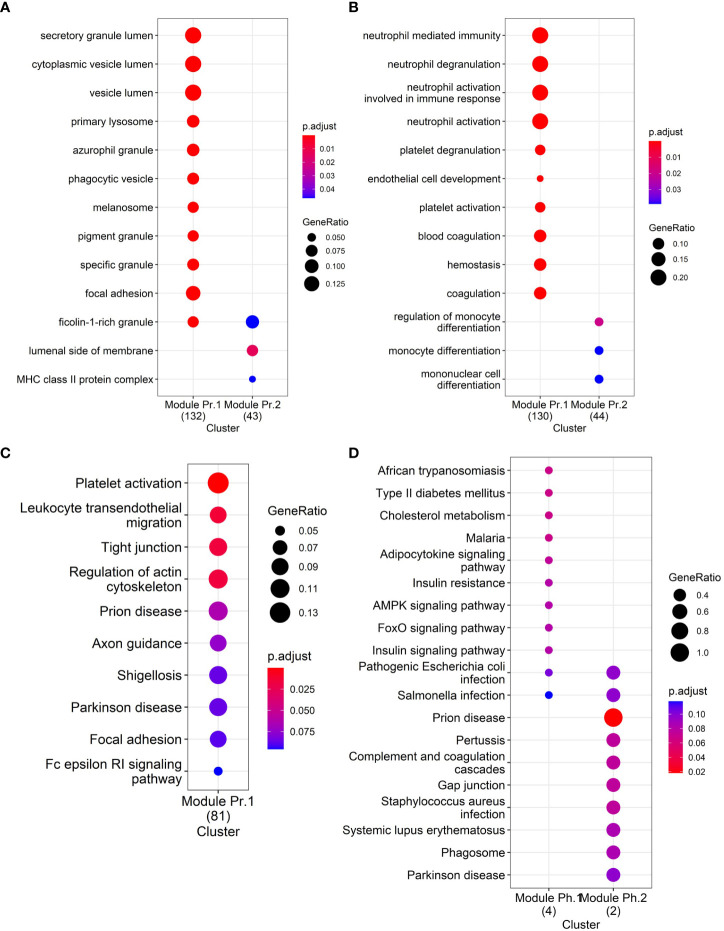
Functional enrichment analyses: **(A)** GO Cellular Compartment enrichment in differentially abundant proteins, **(B)** GO Biological Process enrichment in differentially abundant proteins, **(C)** KEGG pathway enrichment in differentially abundant proteins, and **(D)** KEGG pathway enrichment in differentially abundant phosphoproteins.

As pathway analysis techniques can help in interpreting proteomics results, we applied various enrichment analysis tools to obtain functional information on the differentially abundant proteins. The Gene Ontology (GO) cellular compartment enrichment analysis confirmed that the differentially abundant proteins primarily belong to pathways associated with vesicles and granules: secretory granule lumen, cytoplasmic vesicle lumen, vesicle lumen, membrane raft, membrane microdomain, membrane region indirectly confirming the vesicular origin of the proteins (**Figure 3A**). GO biological process enrichment analysis on the differentially abundant proteins showed that the differentially upregulated proteins are involved in neutrophil degranulation and their activation in immune response, platelet degranulation, blood coagulation, and hemostasis, among others (**Figure 3B**, Module Pr.1). Downregulated EV proteins were enriched in proteins involved in the regulation of monocyte differentiation (**Figure 3B**, Module Pr.2). The Kyoto Encyclopedia of Genes and Genomes (KEGG) pathway analysis also identified platelet activation as one of the most represented pathways together with leukocyte transendothelial migration, and prion-related pathology among others (**Figure 3C**). Interestingly, KEGG pathway analysis of the differentially abundant phosphoproteins also identified a significant enrichment for prion-related pathology (**Figure 3D**).

### 3.4 Weighted gene co-expression network analysis identified key EV-associated protein modules that correlate with pancreas size

To further improve our understanding of the role of circulating EVs in T1D at the system level, we applied the weighted gene co-expression network analysis (WGCNA) methodology for the analysis of all proteins identified in circulating EV-enriched preparations. This method focuses on the identification of modules that include features (i.e., EV-enriched proteins in our case) with correlated expression patterns as opposed to the identification of individual proteins based on group average comparisons (as is the case of differential abundance analysis), therefore alleviating the multiple statistical testing problem. The WGCNA methodology has been previously used for proteomic analysis (26, 27).

**Figure 4:**
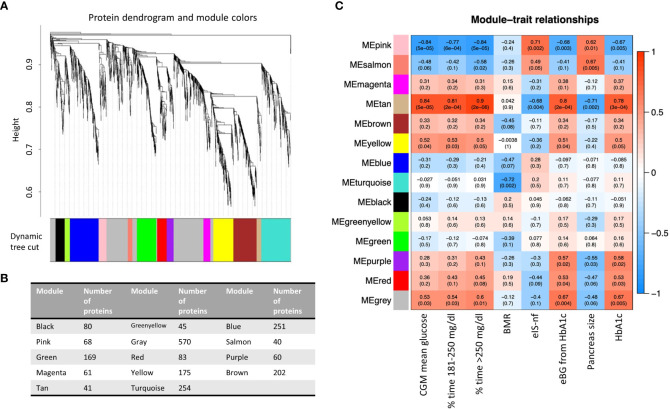
**(A)** Protein dendrogram and module colors. **(B)** Module size. **(C)** Relationships among modules and clinical traits (14 modules 155 clinical traits). Row corresponds to a consensus module. Columns correspond to a trait. Numbers in the table report the correlations of the corresponding module eigengenes and traits, with the p-value below in parenthesis. BMR, basal metabolic rate; eIS-nf, estimated insulin sensitivity with a score including variables not requiring a fasting state; eBG, estimated blood glucose; HbA1c, glycated hemoglobin.

**Figure 4A** presents the co-expression dendrograms for all proteins of EV-enriched preparations and how they were grouped in 14 color-coded modules. **Figure 4B** presents the number of proteins included in each module. Individual proteins of each module can be found in **ST 4**. The analysis identified significant associations of specific protein modules with 9 of the 155 clinical characteristics evaluated in this study. Primarily, the associations were with mean glucose from CGM, percentage of time with glucose >180 mg/dl, HbA1c, blood glucose level estimated from HbA1c, as indicators of glycemic levels, but other interesting traits were identified including an index of insulin sensitivity (eIS-nf), pancreas size and basal metabolic rate. The strongest correlations were seen with clinical traits associated with blood glucose levels (metrics obtained from the CGM, HbA1c) but also with other clinical characteristics indicative of T1D (pancreas size) (**Figure 4C**).

Three co-expression modules (i.e., Pink, Tan, and Yellow modules) were significantly associated with elevated blood glucose levels, expressed as either percentage of CGM values above 250 mg/dl or between 180 and 250 mg/dl, average glucose level during CGM or HbA1 (**Figure 4C**). The list of the 41 proteins selected by connectivity and gene significance of the Tan module is presented in **ST5**. **Supplementary Figure (SF)2** shows the Gene Ontology Biological Processes (GO BP) analysis (panel A) and the heatmap of proteins belonging to relevant pathways identified by the GO BP analysis (panel B) in the Tan module. The Tan module was of particular interest because, in addition to its association with blood glucose levels (correlation 0.9 with % CGM reading >250 mg/dl, p =2E-08), it was associated with pancreas size (correlation -0.71, p=0.002) and estimated insulin sensitivity calculated with parameters not requiring fasting state according to the Coronary Artery Calcification in Type 1 Diabetes (CACTI) study approach (eIS-nf). The eIS calculated using another formula from CACTI that did not include adiponectin levels was not significantly associated with the module.

**Figure 5:**
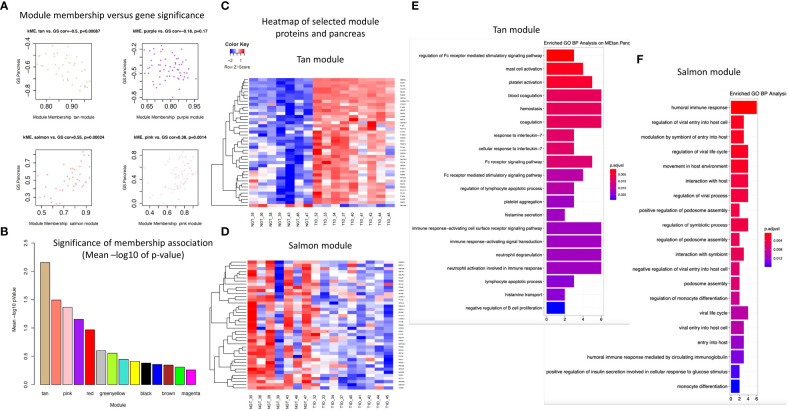
Module connectivity with pancreas size. **(A)** Distribution of module membership versus gene significance. **(B)** Significance of membership association with the trait of interest (pancreas size). **(C)** Heatmap of the EV proteins correlation with pancreas size in the Tan module. **(D)** Heatmap of the EV proteins correlation with pancreas size in the Salmon module. **(E, F)** Enriched GO BP analysis of selected proteins associated with pancreas size in the Tan module **(E)** and in the Salmon module **(F)**.

Four modules (i.e., Tan, Salmon, Pink, Purple, **Figures 4C**, **5A, B, D**) showed significant association with pancreas size that may be a pathogenic clinical feature of T1D (5, 28, 29). Heatmaps of select module proteins (36 of 41 in the Tan module and 19 of 40 in the Salmon module, presented in the **Figures 5C, F**; **ST6** and **ST7**) that displays strong correlations between module membership and protein significance demonstrate the specificity of the EV-enriched protein-pancreas size associations with T1D (**Figures 4C**, **SF4**, **SF5**), particularly for the Tan module proteins. The Pink module also showed a significant correlation between module membership and protein significance (The list of proteins is presented in **ST8**). However, the association was not significant for the Purple module (**Figure 5A**). The GO cellular component enrichment analysis for the Tan module showed a significant enrichment for membrane raft, membrane microdomain, and membrane region compartments as the highest positively correlated components. Among the enriched GO biological processes, several processes associated with inflammation and the immune response were identified (**Figure 5E**). The EV proteins belonging to the Salmon module and associated with pancreas size were enriched for humoral immune response, neutrophil degranulation, regulation of viral entry into host cells, modulation by symbiont of entry into host, regulation of viral life cycle, movement in host environment, interaction with host, regulation of viral processes (**Figures 5F**, **SF4**) that might be relevant in T1D pathogenesis or response to the environment.

**Figure 6:**
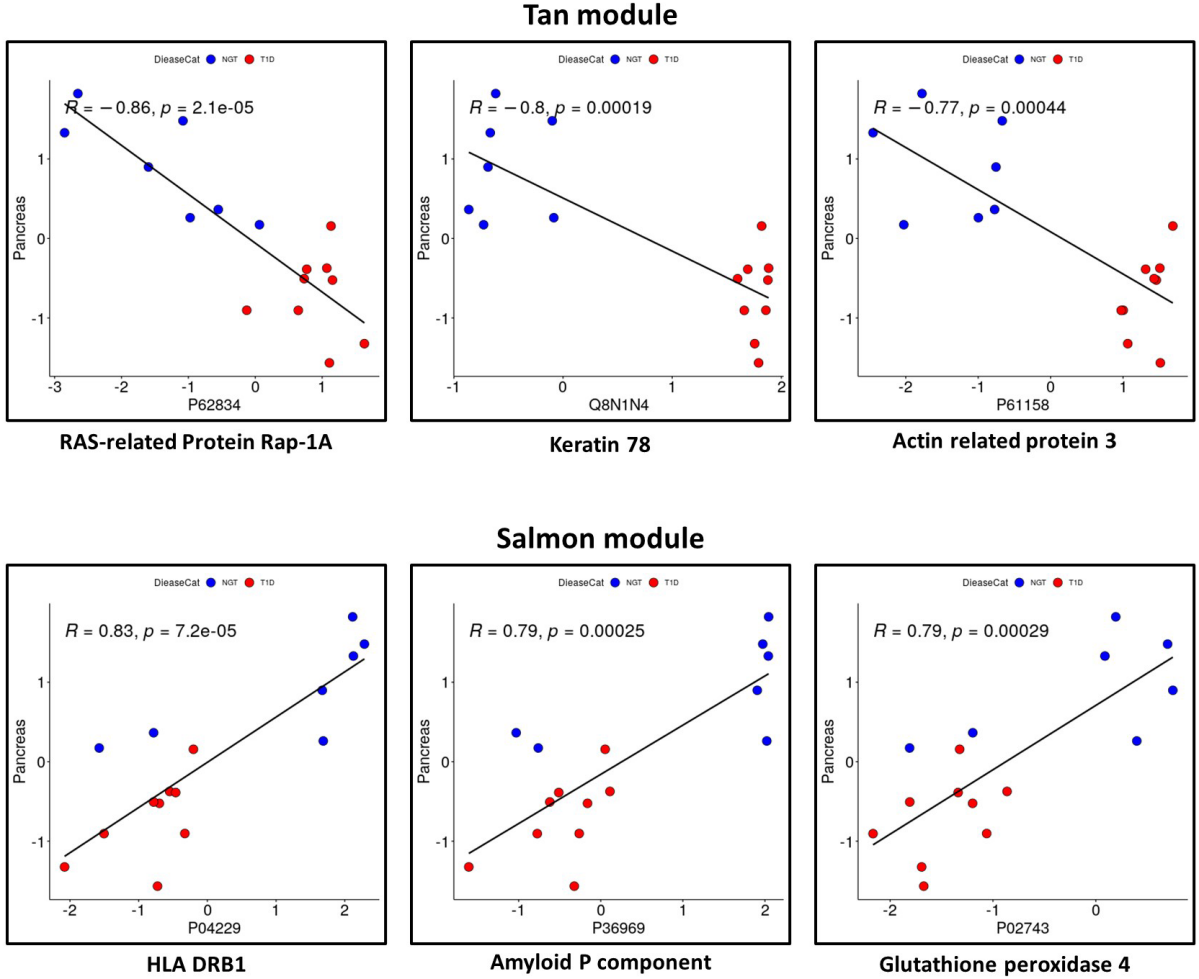
Proteins with a significant correlation with pancreas size in Tan module and in Salmon module. Red: participants with type 1 diabetes (T1D). Blue: participants without diabetes (controls).

Considering the proteins with the strongest correlation with pancreas size, they seemed to be continuously distributed rather than separated in two groups corresponding to T1D and controls (**Figure 6**). This could indicate that the correlation is with the organ size rather than being mediated by a confounder that would discriminate people with diabetes such as glycemic control as possible true markers of disease pathobiology. The Salmon module identified proteins strongly correlated with the clinical trait of interest (i.e., pancreas size) but not appearing among the significantly differentially abundant proteins. One of these, CD74 is an MHC class II chaperon that stabilizes the MHC Class II antigen processing and prevents loading antigen peptides into the MHC Class II complex. CD74 is also involved in the regulation of T-cell and B-cell development, dendritic cell motility, thymic selection, and macrophage inflammation (30). Of note, HLA Class II proteins HLA-DRB1 and HLA-DQB1 are positively associated with pancreas size and are significantly downregulated in the circulating EV-enriched preparations in T1D compared to controls. This supports the concept that more sophisticated analyses such as WGCNA are needed to understand the complex picture of circulating EV-enriched proteins.

Some of the modules (Green, Brown, Turquoise, Yellow) correlated with the mode of particle size of EV-enriched preparations (Green corr=0.6, p=6.7e-18, Brown cor=0.22, p=0.0017, turquoise cor=0.37, p1.2e-09, yellow cor=0.34, p=4.2e-06). This is expected as different types of EVs have different sizes and cargos. Significant correlations were revealed between member proteins in these modules and particle size mode but not associated with disease status. The network module association with key diabetes indicators like HbA1c, glucose measurements, and pancreas size was clearly different than the association with EV/particle size.

## 4 Discussion

This pilot study showed that the proteome of circulating EV-enriched preparations of patients with T1D differs from the one of healthy volunteers and significantly correlates with key features of T1D. Our proteomic and phosphoproteomic analyses demonstrated significant enrichment in annotated exosomal proteins and phosphoproteins. This suggests that our study preparations were enriched in exosomes. The EV cargo is reported to mirror, to some extent, the specific cellular phenotypes from their cells of origin (31). Therefore, it could provide information on mechanisms that are active in the disease of interest. To our knowledge, this is the first characterization of the proteome of circulating EV-enriched preparations in patients with T1D compared to controls. The plasma or serum proteome includes a heterogenous pool of proteins from vesicles derived by multiple cell types, with production driven by multiple mechanisms (32, 33). We were not surprised to find that the proteome and phosphoproteome of EV-enriched preparations were associated with hyperglycemia, insulin treatment and glucose variability as these are key drivers of T1D pathophysiology, but also with T1D pathogenic mechanisms. The objective of this work was to verify that potential pathogenic mechanisms were not entirely masked by glucose driven signatures. The results of the present investigation will guide future studies as these relationships may be important for discriminating T1D endotypes that may respond differently to the now available preventive therapies for T1D. On the other hand, EV-enriched preparations could indicate pathways that could eventually identify mechanisms leading to progression to chronic complications of the disease which will require a more aggressive monitoring and treatment.

Supporting the information richness and biomarker potential of circulating EV-enriched preparations, our bioinformatic approaches were able to discriminate the two study populations, namely people with T1D and controls, and to identify key proteins that represent known and novel aspects of T1D biology. By analyzing cellular compartment annotations for the differentially abundant proteins and phosphoproteins, we demonstrated enrichment in vesicle and secretory granule associated proteins likely involved in insulin, platelet, and neutrophil function. This leads us to the hypothesis that T1D could indeed modulate EV biogenesis and secretion. As expected, biological process and pathway analysis identified enrichment for pathways (e.g., neutrophil degranulation and activation) that may be associated not only with the pathogenesis of the disease but also with the development of chronic complications, both at the macro and microvascular level. Pathways connected to platelet function and coagulation suggest the possible utility of EV proteomics in the prediction of disease complications. Specifically, C1q and plasminogen (PLG) were two of the key complement proteins strongly increased in our T1D EV-enriched preparations. Notably, deposition of complement proteins like C1q in the EVs has been reported to be needed for complement activation and, when deregulated, to contribute to pathological states (34). These particles loaded with complement regulators are suggested to contribute to the stimulation and inhibition of T-cell responses and the interaction among immune cells (35). Furthermore, C1q has been recognized as an important pattern recognition receptor that diverts autoantigen containing extracellular vesicles from immune recognition (36). PLG, on the other hand, is reported to be more efficiently activated by PLG activators when associated with cell surfaces, as compared to the reaction in solution (37). By the same token, PLG on vesicle surfaces may be more efficiently converted into plasmin, consequently promoting fibrinolysis and immune cell modulation with systemic effects.

Additionally, 8 of the top 100 ExoCarta proteins were found to be significantly upregulated in the T1D circulating EVs (CD63, RAB14, VCP, BSG (a.k.a. CD147), FLNA, GNAI2, LAMP2, EZR) and to significantly correlate with clinical measures of glycemic control, insulin sensitivity, and pancreas size. Because the ExoCarta database is a heterogenous aggregator of published proteins presumably enriched in exosomal preparations but still includes contaminants that copurify with EVs isolated by standard methods, we focused on the subset of 100 proteins most often identified in exosome studies and catalogued in the database [http://exocarta.org/exosome_markers_new (25)]. A number of publications indicated that CD63, RAB14, BSG, LAMP2, EZR are transmembrane proteins confirmed to be present in EV preparations obtained with different methods of isolation which should reduce the effect of contamination (38–41). The WGCNA revealed significant correlations between member proteins in some of the modules and particle size mode but they were not associated with disease status. Further studies with larger sample sizes are necessary to clarify if T1D modulates EV biogenesis and secretion and/or that dysregulation of EV-mediated processes may contribute to T1D development and/or progression.

Among other proteins involved in the inflammatory response and immune-activation we found upregulated IL6ST/Gp130 (the founding member of the cytokine receptor family, which is involved in the regulation of adipocyte development and function and has a key role at the intersection of inflammation, autoimmunity and cancer) (42, 43) and CD40, and the downregulated MHC class II proteins HLA-DQB1 and HLA-DRB1. Of note, all these proteins are involved in IL-6 signaling (42–48). Importantly, and supporting our T1D-related findings, it is known that macrophages from T1D patients with high-risk HLA-DQB1 alleles are sensitized to secrete IL-6 in response to nonantigenic stimulation (46). Interestingly, phosphorylated MARCH8 (gene symbol: MARCHF8), an E3 ubiquitin-protein ligase that regulates the expression and trafficking of critical immunoreceptors such as the MHC class II proteins (49, 50), specifically in non-hematopoietic cells (i.e., thymic and alveolar epithelial cells) (51), was also significantly downregulated in the circulating EV-enriched preparations.

In addition to the neutrophil, platelet, and immune cell activation related pathways, the differentially abundant proteins and phosphoproteins were enriched for other biological processes and KEGG pathways related to prion disease. In this context, it is of interest to highlight that Wasserfall’s group (52) recently showed an increased frequency of cellular prion proteins in pancreas specimens from people with T1D and that, earlier, Strom and collaborators (53) demonstrated in two rodent models that altered metabolism of cellular prion protein in β cells associated with glucose dysregulation. Interestingly, we detected a significantly elevated phosphoprotein/total protein ratio for nucleobindin-1 (NUCB1) in the circulating EV-enriched preparations from the T1D participants. NUCB1 is a calcium-binding protein of the Golgi with a hypothetical role in calcium homeostasis (54). Some of its described functions may be relevant for T1D pathogenesis. NUCB1 is an ER stress-inducible gene (55) expressed in numerous tissues. It is expressed in the pancreatic islets but not in acinar cells (56), and in its soluble form it inhibits amyloid fibril deposition including hIAPP (57). It is overexpressed in Lupus and is a B-cell differentiation and growth factor. The significantly elevated NUCB1 phosphoprotein/protein ratio in the preparations is due to a significant downregulation at the protein level while the phosphorylation status of the protein was not significantly elevated. We reason that the reduction of nucleobindin-1 protein in EVs, which additionally appear to be present in an altered phosphorylation status, may indicate that nucleobindin-1 function (possibly in both the cell and the released EVs) is impaired (reduced) in the pancreas of T1D patients. This impairment in NUCB1 protein function may contribute to the accumulation of amyloid fibrils in pancreatic islets. We further identified, using the WGCNA methodology, that Salmon module protein serum amyloid P-component (SAP/APCS, an acute phase inflammation protein found in the circulation) correlated with pancreas size. Similar to NUCB1, SAP was also found to be significantly downregulated in our T1D participants. Interestingly, SAP and hs-CRP (another acute phase inflammation protein) were recently reported to show opposite and synergic associations with all-cause mortality in patients with type 2 diabetes (58), however, the protective role of SAP on the risk of mortality is not well understood. Amyloid deposition has been described in pancreata of T1D patients (59, 60).

Taking advantage of the deep metabolic phenotyping of our participants, we attempted at more precisely characterize the proteome of EV-enriched preparations and its association with clinical features (beyond the analysis of differential expression based on group averages comparisons) by applying a WGCNA methodology. Coexpression networks and WGCNA identify clusters of genes based on their correlated expression patterns (19). This approach can find potential novel candidates based on co-expression similarities, rather than protein-protein interaction that may have limitations (61). The literature on the adaptation of the WGCNA methodology on proteomic data is relatively limited and only applied to urinary EV (62).

WGCNA applied to proteomics of EV enriched preparations in our study identified 14 distinct modules significantly associated with nine clinical features. As expected, the association is primarily with blood glucose measures indicative of T1D, but also with other features like pancreas size, resting energy expenditure, and the CACTI index of insulin sensitivity. The enrichment analysis of gene ontologies pointed to the biological meaning of the modules, identifying neutrophil activation and degranulation, activation of immune response and blood coagulation as some of the pathways associated with hyperglycemia, which were also identified by the differential abundance analysis. Importantly, the humoral immune response, regulation of viral entry and life cycle regulation of viral processes were associated with pancreas size in the Salmon module together with mast cell activation, platelet activation, blood coagulation, hemostasis, and response to IL-7 in the Tan module. As hyperglycemia is the characteristic feature of diabetes it is impossible to discriminate in this cross-sectional study whether the proteins included in these modules are differentially abundant as a cause or a consequence of hyperglycemia. However, features possibly linked to pathogenic mechanisms, are also identified.

The WGCNA methodology has been applied to peripheral blood gene expression in T1D and controls (63, 64) and identified significantly disrupted co-expression modules in T1D compared to healthy control PBMCs (63). The module associated with T1D in the work of Lu et al. (63) was enriched for genes belonging to the “regulation of immune response” pathway. Our study took a broader approach considering proteomics of circulating EV-enriched preparations. Circulating EVs originate from different cells, therefore, the modules that we identified are likely a composite of coregulated proteins expressed from a number of different cellular sources, including PBMCs. In line with what was seen in PBMC transcriptomics (63), we also identified enrichment in proteins classified under the broad biological process ontology denominated “regulation of immune response” (that includes 1019 genes) and associated with hyperglycemia as an indicator of diabetes (regulation of Fc receptor mediated stimulatory signaling pathway). In our approach, we include proteomic profiles obtained from patients with T1D and healthy controls to identify clinically relevant protein clusters (modules) that correlate with clinical variables of interest, independently of the disease. Therefore, the proteins included in the modules of interest could be used as markers of that clinical variable rather than of the disease. The analysis focused then on the association with pancreas size, as a trait that might be associated with disease pathogenesis opening the avenue for confirmatory studies in new onset diabetes or precious islet autoantibody positive samples.

Among the proteins of interest identified by our traditional (differential expression) approach and by WGCNA, HLA-DRB1 and DQB1 were significantly downregulated and correlated with pancreas size. MHC-Class II molecules are present in exosomes originating from APC, B-cells and T-cells (65–67). Consistent with our findings, MHC Class II-loaded EVs are also detected in plasma (68). The MHC Class II in EVs is the predominant form of MHC proteins detected in serum and plasma, even though a soluble form has also been described. Both the soluble MHC-Class II and the exosome-associated MHC-Class II are immunomodulatory (69). Immunosuppressive EVs derived from APCs occur naturally, for example, soon after eating or inoculation of specific antigens (70). The experiments by Kim and collaborators (68) suggested that the immune response to a foreign antigen is regulated by exosomes in plasma produced by monocytes/macrophages that have the ability to suppress the immune response in an antigen-specific manner. Therefore, we could hypothesize that lower levels of MHC in EV-enriched preparations in T1D compared to healthy individuals could be a specific sign of the loss of tolerance in T1D. Our study was not able to demonstrate that this mechanism is active in T1D pathogenesis, however, it highlighted the power of WGCNA in analyzing complex diseases and in formulating hypotheses. Paired with the in-depth phenotyping of the participants of this pilot study, WGCNA allowed us to identify and confirm the potential role of selected proteins that would have been excluded by simple differential expression analysis.

The primary limitation of our study is the small sample size, therefore the conclusions cannot be generalizable and might have missed important pathways. The power of the applied methodology is that it produces meaningful results even with a small sample size as in our case. The gene connectivity analysis smooths the variability and alleviates the need for multiple sampling. The cross-sectional design needs to be considered hypothesis generating and the findings should be confirmed in larger and independent cohort(s), and ultimately in dedicated functional studies including early stages of T1D development.

## 5 Conclusions

Proteomics of circulating EV-enriched preparations appear to be a powerful tool to highlight potential disease biomarkers that could be the object of validation studies. WGCNA adds the opportunity to identify specific clinical phenotypes that could be investigated in future mechanistic studies.

## Data availability statement

The original contributions presented in the study are publicly available. This data can be found here: ProteomeXchange Consortium *via* the PRIDE partner repository with the dataset identifier PXD041899 (71).

## Ethics statement

The studies involving human participants were reviewed and approved by AdventHealth Institutional Review Board. The patients/participants provided their written informed consent to participate in this study.

## Author contributions

AC designed and supervised experiments, researched data, and wrote the manuscript; YNL conducted experiments, researched and analyzed data, and wrote the manuscript; GY researched and analyzed data and contributed to manuscript writing; CC and ABh researched data and contributed to manuscript editing; ABi, contributed to clinical study execution and researched data and contributed to manuscript editing, AP, HC, and KC conducted experiments and contributed to manuscript editing; AI conducted experiments, researched data, and contributed to manuscript editing; DM designed the larger clinical study, acquired funding, and reviewed the manuscript, RP designed the study, acquired funding, provided the study samples, supervised the work, and reviewed/edited the manuscript. All authors contributed to the article and approved the submitted version.
