# Supplementary material for: The proteome and phosphoproteome of circulating extracellular vesicle-enriched preparations are associated with characteristic clinical features in type 1 diabetes
Source: Front Endocrinol (Lausanne). 2023 Jul 28;14:1219293. doi: 10.3389/fendo.2023.1219293 (PMC10417723; doi:10.3389/fendo.2023.1219293)

Supplementary Figure 1: Significance testing of module associations with relevant clinical characteristics.
Panel A reports the mean -log10 of the p-value of the association of proteins of each module with % of time >250 mg/dl measured with CGM.
Panel B reports the mean -log10 of the p-value of the association of proteins of each module with Pancreas size. (The graph is also reported in Figure 5 and presented here for completeness).
The graphs below guided the selection of modules that underwent a deeper analysis.

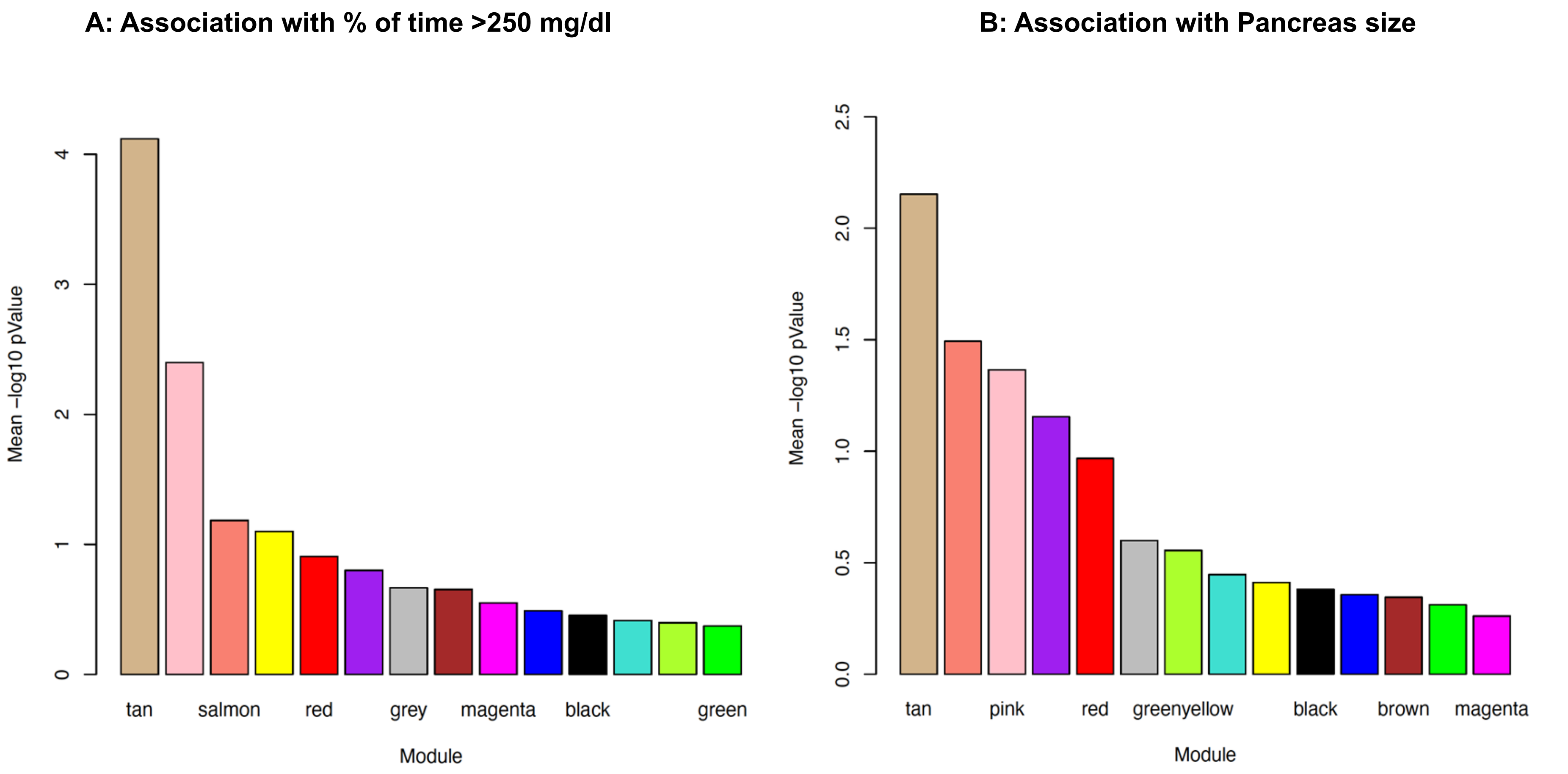

Supplementary Figure 2

A) Enriched GO BP analysis and B) Enriched GO CC analysis of 41 proteins significantly associated with % of time > 250mg/dl in the Tan module.

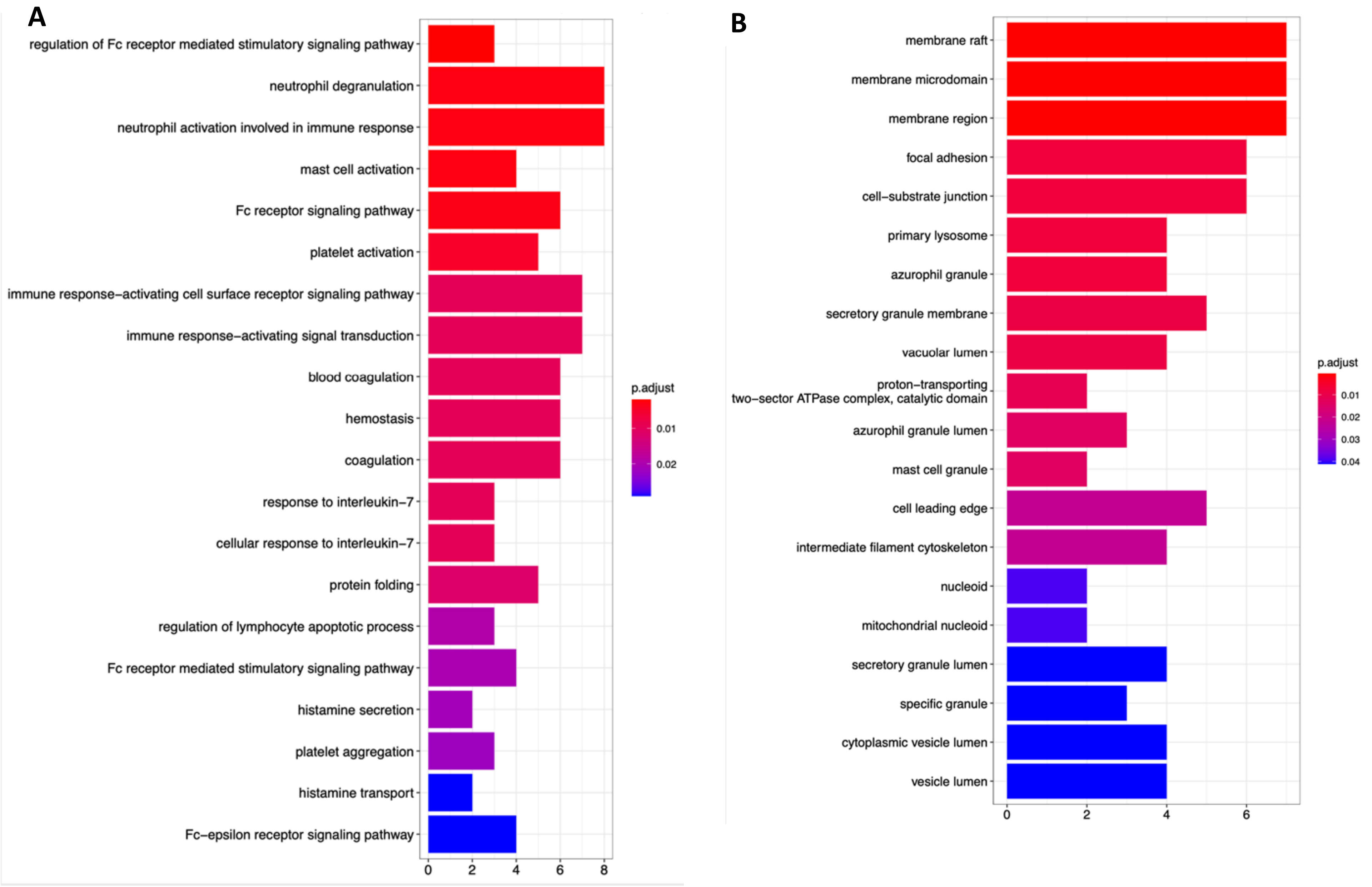

Supplementary Figure 3

Heatmaps indicating the association and significance of proteins in the Tan module with % time > 250 mg/dl in participants with and without T1D belonging to the A) GO BP pathway Neutrophil activation involved in immune response, B) GO BP pathway Mast cell activation, C) GO CC pathway Secretory granule membrane, D) Primary lysosome.

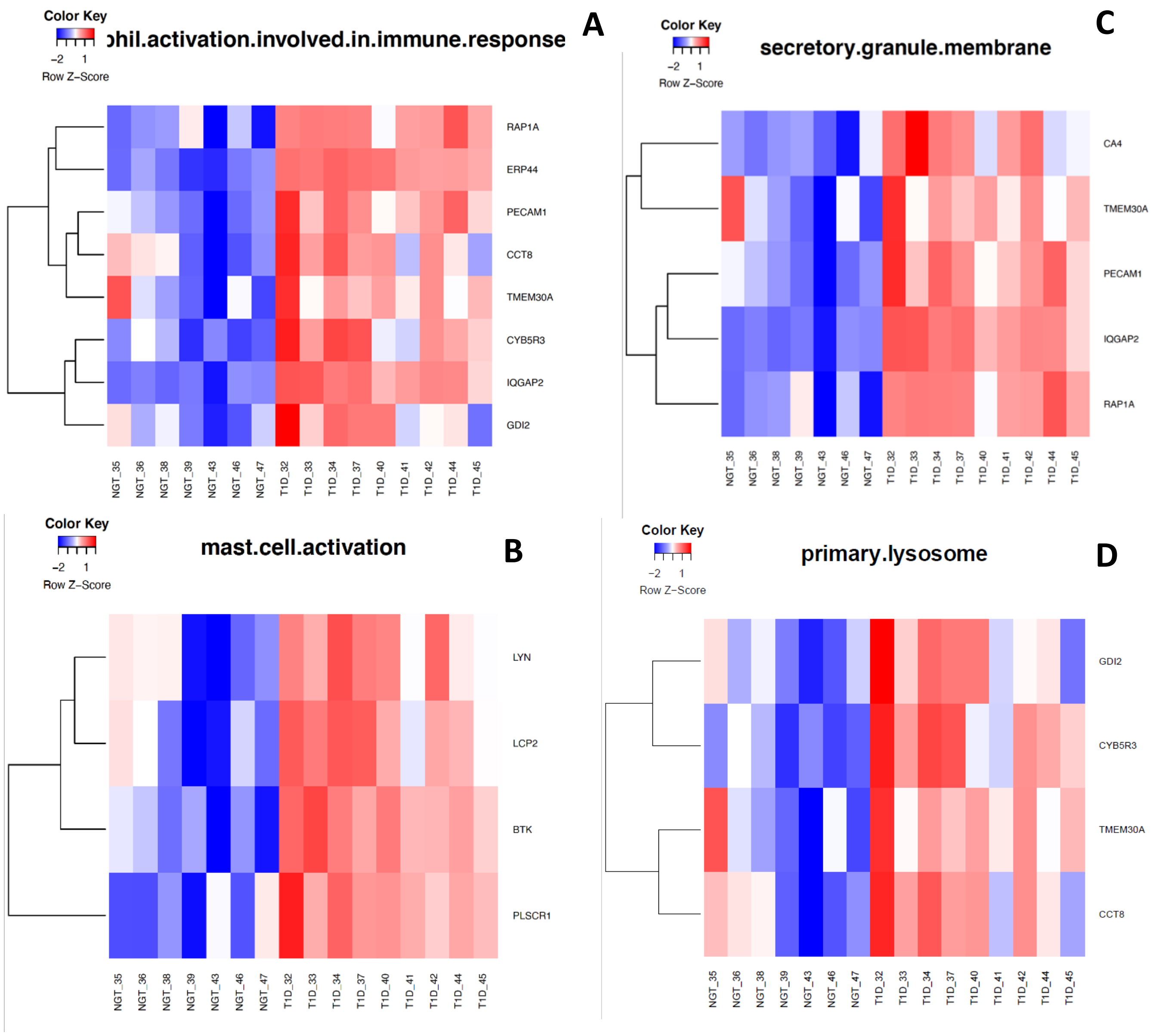

Supplementary Figure 4

A) Enriched GO CC analysis of 36 proteins significantly associated with pancreas size in the Tan module. B) Heatmap indicating the association and significance of proteins of the Tan module belonging to the GO CC pathway Membrane microdomain and pancreas size in participants with and without T1D.

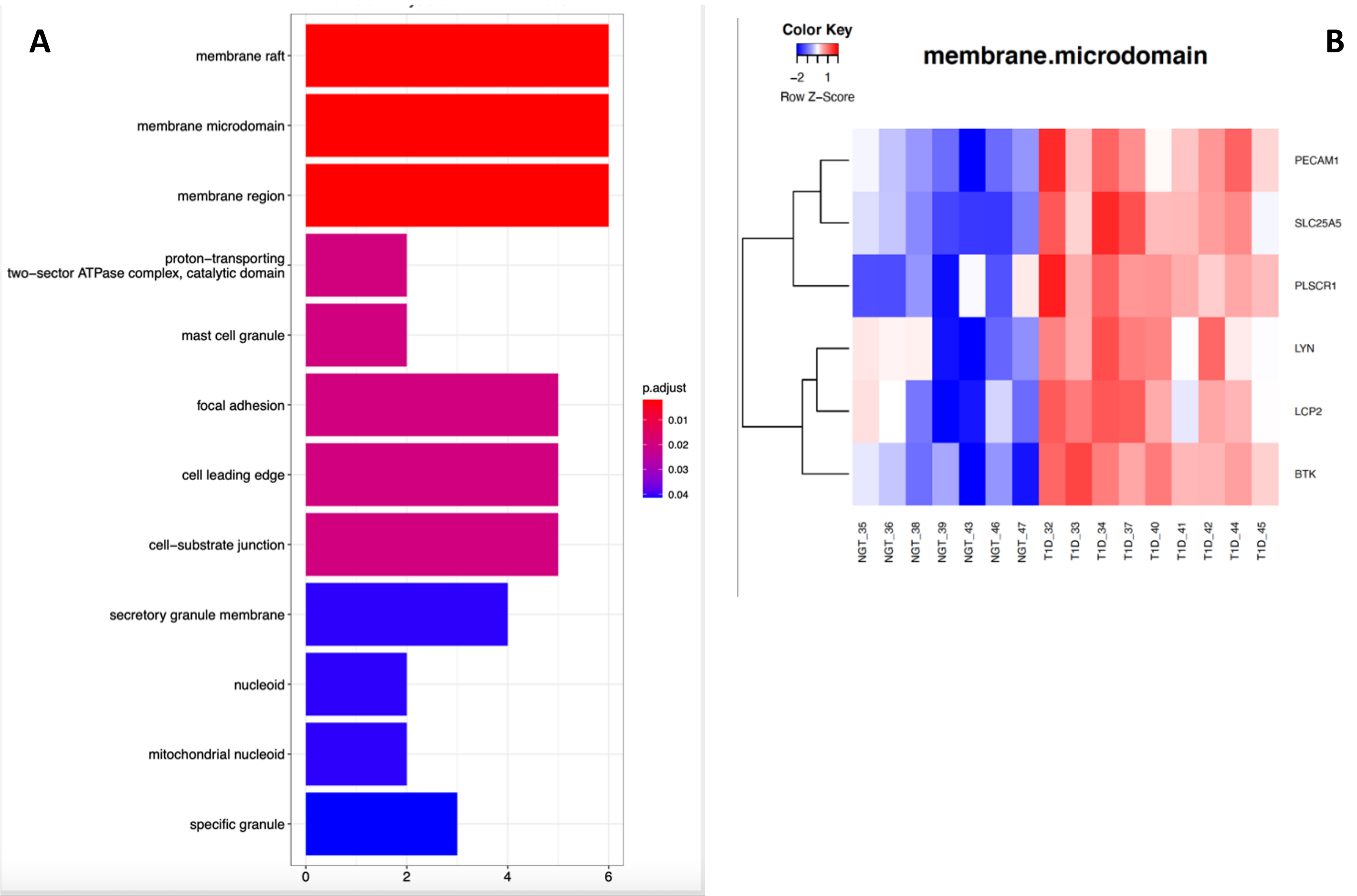

Supplementary Figure 5

Heatmap indicating the association and significance of proteins of the Tan module belonging to the GO BP pathway Neutrophil degranulation and pancreas size in participants with and without T1D.

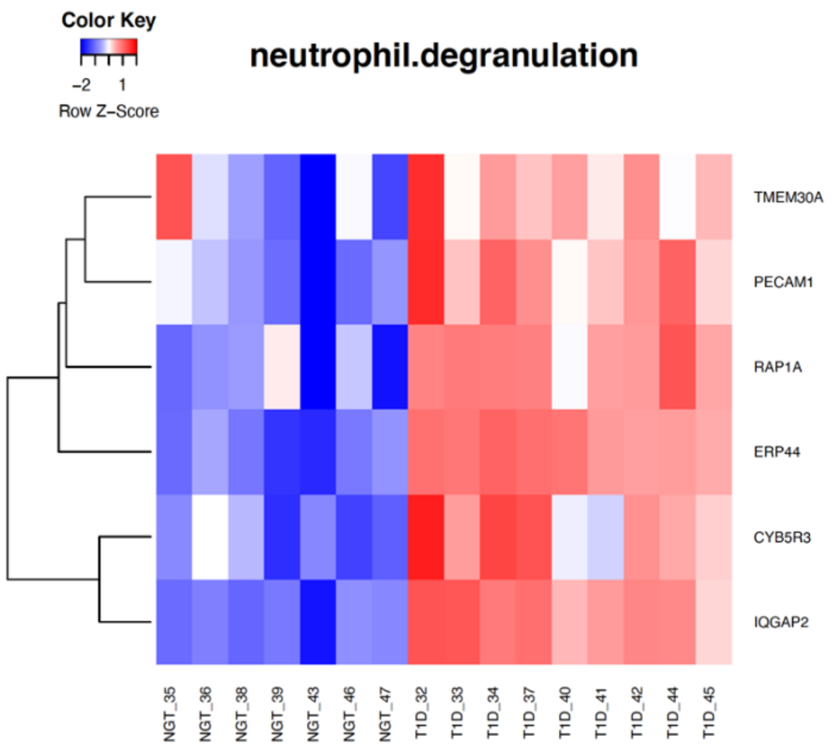

Supplementary Figure 6

Heatmap indicating the association and significance of proteins of the Salmon module belonging to the GO BP pathway Humoral immune response and pancreas size in participants with and without T1D.

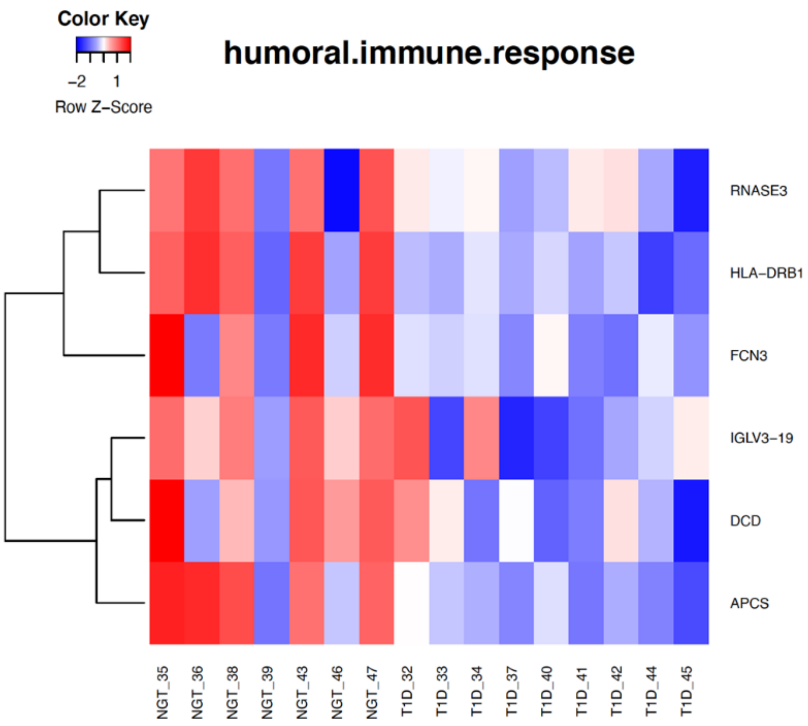

Supplement: Supplementary file 1 [file Image_1.pdf]
