# Supplementary material for: The proteome and phosphoproteome of circulating extracellular vesicle-enriched preparations are associated with characteristic clinical features in type 1 diabetes
Source: Front Endocrinol (Lausanne). 2023 Jul 28;14:1219293. doi: 10.3389/fendo.2023.1219293 (PMC10417723; doi:10.3389/fendo.2023.1219293)
Supplement: Supplementary file 2 [file Table_1.docx]

**Supplementary Table ST1**: Clinical characteristics of the study cohort not reported in Table 1

| Variable | Control | T1D | p |
| --- | --- | --- | --- |
| N | 7 | 10 |  |
| Height_Avg (cm ) | 168.60 [152.02, 171.15] | 163.50 [158.40, 168.50] | 0.696 |
| Weight_avg (kg) | 62.90 [54.95, 88.46] | 73.35 [63.15, 79.07] | 0.77 |
| Mean systolic blood pressure (mmHg ) | 120.50 [111.50, 124.25] | 126.25 [114.88, 132.00] | 0.241 |
| Mean diastolic blood pressure (mmHg) | 66.50 [62.75, 78.50] | 76.00 [71.00, 79.38] | 0.329 |
| Mean heart rate (bpm) | 62.50 [59.00, 67.00] | 75.25 [69.00, 83.12] | 0.011 |
| Waist_average (cm) | 78.25 [75.08, 95.50] | 86.38 [78.72, 92.25] | 0.696 |
| Hip_average (cm) | 102.00 [85.75, 103.22] | 95.12 [91.96, 102.67] | 0.922 |
| Waist_Hip_Ratio (cm) | 0.86 [0.81, 0.89] | 0.86 [0.84, 0.90] | 0.695 |
| Albumin (g/dL) | 4.60 [4.35, 4.70] | 4.45 [4.32, 4.50] | 0.3 |
| Alkaline Phosphatase (U/L) | 54.00 [49.50, 62.50] | 70.00 [62.75, 87.50] | 0.04 |
| ALT (U/L) | 16.00 [13.00, 24.50] | 15.00 [11.75, 16.00] | 0.524 |
| AST (U/L) | 24.00 [22.00, 26.50] | 20.00 [18.25, 22.00] | 0.171 |
| BilirubinTotal (mg/dL) | 0.40 [0.30, 0.45] | 0.55 [0.43, 0.75] | 0.029 |
| BUN (mg/dL) | 11.00 [10.00, 13.00] | 12.00 [11.00, 12.75] | 0.73 |
| Creatinine (mg/dL) | 0.74 [0.70, 0.85] | 0.76 [0.68, 0.83] | 0.922 |
| Glomerular Filtration Rate by race (CKD.EPI) (mL/min) | 122.00 [108.50, 131.00] | 119.50 [116.75, 124.50] | 1 |
| Globulin (g/dL) | 2.80 [2.60, 3.25] | 3.00 [2.73, 3.25] | 0.589 |
| Glucose (mg/dL) | 90.00 [86.00, 93.50] | 142.00 [120.50, 199.50] | 0.04 |
| Total Proteins (g/dL) | 7.40 [7.30, 7.90] | 7.20 [7.10, 7.60] | 0.327 |
| Absolute_Basophil_Count (cells/uL) | 0.04 [0.03, 0.06] | 0.04 [0.04, 0.06] | 0.446 |
| Abolute_Eosinophil_Count (cells/uL) | 0.13 [0.07, 0.40] | 0.11 [0.09, 0.19] | 0.769 |
| Absolute_Lymphocyte_Count (cells/uL) | 2.01 [1.73, 2.50] | 1.54 [1.39, 1.99] | 0.13 |
| Absolute_Monocyte_Count (cells/uL) | 0.37 [0.34, 0.54] | 0.42 [0.36, 0.49] | 0.922 |
| Absolute_Neutrophil_Count (cells/uL) | 3.85 [2.80, 5.40] | 3.74 [2.84, 4.78] | 0.922 |
| Basophils % | 0.50 [0.50, 0.85] | 0.70 [0.60, 0.98] | 0.183 |
| Eosinophils % | 1.60 [1.25, 5.95] | 1.65 [1.35, 3.38] | 0.883 |
| Hematocrit % | 41.10 [37.65, 43.85] | 41.55 [39.42, 44.35] | 0.696 |
| Hemoglobin (g/dL) | 13.00 [11.85, 14.55] | 13.80 [13.30, 14.92] | 0.328 |
| Lymphocytes % | 33.60 [26.30, 38.40] | 29.10 [27.55, 30.20] | 0.435 |
| Monocytes % | 5.70 [5.55, 7.40] | 7.30 [7.10, 7.68] | 0.24 |
| Neutrophils % | 56.80 [50.40, 64.60] | 61.30 [57.15, 62.50] | 0.525 |
| Platelet_Count (cells/uL) | 280.00 [248.50, 290.50] | 290.00 [254.25, 322.00] | 0.77 |
| RBC (cells/uL) | 4.46 [4.39, 5.08] | 5.00 [4.58, 5.22] | 0.261 |
| WBC (cells/uL) | 6.53 [5.44, 8.71] | 6.24 [4.88, 7.01] | 0.495 |
| TSH mIU/mL) | 1.80 [1.27, 2.21] | 1.95 [1.39, 2.69] | 0.626 |
| Cholesterol (mg/dL) | 159.00 [141.00, 168.50] | 142.50 [126.75, 167.00] | 0.329 |
| Chol-HDL_Ratio | 2.60 [2.40, 4.20] | 2.40 [2.22, 3.05] | 0.305 |
| HDL (mg/dL) | 63.00 [45.00, 64.50] | 55.50 [44.00, 68.25] | 0.961 |
| LDL (mg/dL) | 84.00 [79.50, 85.50] | 72.00 [63.75, 86.00] | 0.222 |
| LDL/HDL_Ratio | 1.30 [1.20, 2.60] | 1.20 [1.10, 1.78] | 0.326 |
| NonHDL-Cholesterol (mg/dL) | 100.00 [96.00, 107.00] | 82.50 [74.75, 95.50] | 0.107 |
| Triglycerides | 87.00 [53.50, 133.00] | 54.50 [45.50, 87.00] | 0.157 |
| VLDL (mg/dL) | 17.00 [11.00, 26.50] | 11.00 [9.00, 17.00] | 0.171 |
| %_Liver_Fat (PDFF) | 1.25 [0.94, 4.96] | 1.17 [1.10, 1.59] | 0.77 |
| MRE_Average_Stiffness (kPa) | 1.94 [1.81, 2.17] | 1.85 [1.79, 2.15] | 0.596 |
| SAT_sum Area Mid Femur Slice (cm^2) | 13069.77 [11578.63, 25190.13] | 18179.54 [13231.18, 21877.71] | 0.558 |
| IMAT_sum Area Mid Femur Slice (cm^2) | 1783.27 [1374.26, 3073.03] | 2059.82 [1268.76, 2771.35] | 0.922 |
| Muscle_sum Area Mid Femur Slice (cm^2) | 24363.55 [22914.31, 32462.90] | 26777.19 [23849.12, 29230.38] | 0.922 |
| Bone_sum Area mid femur slice (cm^2) | 979.34 [829.33, 1220.66] | 1052.55 [908.29, 1171.28] | 0.696 |
| SAT_sum Volume (cm^3) | 1103042.32 [981185.76, 2087630.16] | 1521280.91 [1113009.38, 1841803.88] | 0.626 |
| IMAT_sum Volume (cm^3) | 170450.85 [127699.39, 274042.90] | 190781.10 [107734.79, 255509.14] | 0.845 |
| Muscle_sum Volume (cm^3) | 1950434.18 [1846165.17, 2626980.61] | 2151890.38 [1941567.85, 2364076.91] | 0.922 |
| Bone_sum Volume (cm^3) | 81232.92 [69983.96, 100696.56] | 87545.61 [75487.66, 96750.76] | 0.696 |
| VAT Mass_DEXA (cm^3) | 162.80 [123.46, 626.84] | 306.31 [255.11, 771.87] | 0.626 |
| VAT Volume_DEXA (cm^3) | 172.56 [130.86, 664.45] | 324.69 [270.42, 818.18] | 0.626 |
| Total Fat Mass (kg) | 21.858 [18.451, 33.384] | 24.440 [18.852, 28.248] | 0.77 |
| Total Lean Mass (kg) | 36.569 [34.872, 54.471] | 43.525 [40.360, 53.199] | 0.435 |
| Total Fat-Free Mass (kg) | 39.285 [36.945, 57.863] | 46.038 [42.653, 55.947] | 0.435 |
| Total Tissue Mass (kg) | 60.76 [53.49, 86.00] | 71.81 [60.48, 77.00] | 0.77 |
| Total Total Mass (kg) | 62.94 [55.80, 89.40] | 74.34 [62.62, 80.13] | 0.77 |
| Arms Region - Fat proportion | 0.32 [0.27, 0.40] | 0.34 [0.25, 0.38] | 0.845 |
| Legs Region - Fat proportion | 0.37 [0.31, 0.41] | 0.36 [0.32, 0.43] | 0.845 |
| Trunk Region - Fat proportion | 0.34 [0.30, 0.43] | 0.35 [0.29, 0.38] | 0.558 |
| Android Region - Fat proportion | 0.36 [0.32, 0.46] | 0.36 [0.27, 0.41] | 0.626 |
| Gynoid Region - Fat proportion | 0.37 [0.31, 0.41] | 0.35 [0.33, 0.47] | 0.845 |
| Total Region - Fat proportion | 0.34 [0.29, 0.41] | 0.35 [0.28, 0.39] | 0.845 |
| Arms Tissue - Fat proportion | 0.33 [0.29, 0.41] | 0.35 [0.26, 0.39] | 0.845 |
| Legs Tissue - Fat proportion | 0.38 [0.32, 0.42] | 0.38 [0.33, 0.44] | 0.922 |
| Trunk Tissue - Fat proportion | 0.35 [0.31, 0.44] | 0.36 [0.29, 0.39] | 0.558 |
| Android Tissue - Fat proportion | 0.37 [0.32, 0.46] | 0.36 [0.27, 0.42] | 0.626 |
| Gynoid Tissue - Fat proportion | 0.38 [0.32, 0.42] | 0.36 [0.34, 0.48] | 0.77 |
| Total Tissue - Fat proportion | 0.36 [0.30, 0.42] | 0.36 [0.30, 0.40] | 0.77 |
| Energy intake measured (Kcal/day) | 1765.00 [1656.00, 2497.00] | 2075.00 [1875.50, 2329.75] | 0.626 |
| Energy intake predicted (Kcal/day) | 1969.00 [1772.00, 2389.50] | 2161.50 [1890.50, 2323.25] | 0.495 |
| 24-Hour Energy Expenditure (Kcal/day) | 1767.00 [1594.00, 2531.50] | 2128.00 [1814.75, 2259.50] | 0.77 |
| 24Hour RER | 0.88 [0.87, 0.89] | 0.89 [0.87, 0.91] | 0.66 |
| Exercise_EE (kcal/min) | 3.66 [3.50, 5.46] | 4.53 [3.98, 4.64] | 0.848 |
| Exercise_RER | 0.92 [0.92, 0.93] | 0.92 [0.90, 0.97] | 0.949 |
| Sleeping metabolic rate (SMR) (kcal/day) | 1255.00 [1151.50, 1805.00] | 1501.00 [1347.50, 1720.50] | 0.696 |
| Sleeping metabolic rate _RER | 0.86 [0.84, 0.87] | 0.86 [0.84, 0.88] | 1 |
| 24Hour EE (Non-Exercise Non-Sleep) (kcal/day) | 1804.00 [1617.50, 2527.00] | 2163.50 [1958.25, 2268.00] | 0.495 |
| 24Hurs RER (Non-Exercise Non-Sleep) | 0.89 [0.88, 0.89] | 0.90 [0.87, 0.92] | 0.495 |
| Non-protein RER | 0.89 [0.88, 0.91] | 0.90 [0.88, 0.92] | 0.77 |
| Carbohydrate Oxidation (g/day) | 248.00 [205.50, 362.00] | 283.50 [246.75, 336.25] | 0.696 |
| Fat Oxidation (g/day) | 59.00 [47.00, 66.50] | 61.00 [40.75, 77.25] | 0.626 |
| Protein Oxidation (g/day) | 80.00 [60.50, 99.00] | 65.00 [57.00, 79.25] | 0.464 |
| Spontaneous physical activity (SPA – min/day) | 54.00 [32.50, 74.50] | 76.50 [60.75, 102.50] | 0.187 |
| Thermic effect of food (%) | 0.04 [0.03, 0.06] | 0.06 [0.03, 0.07] | 0.922 |
| Resting metabolic rate (pre-breakfast) (kcal/day) | 1516.00 [1380.50, 1808.50] | 1609.00 [1528.25, 1851.25] | 0.494 |
| RER_pre-breakfast | 0.86 [0.83, 0.86] | 0.87 [0.85, 0.87] | 0.283 |
| Resting metabolic rate (post-breakfast) (kcal/day) | 1571.00 [1440.00, 1977.50] | 1743.00 [1616.25, 1914.75] | 0.495 |
| RER (post-breakfast) | 0.90 [0.88, 0.92] | 0.92 [0.91, 0.94] | 0.143 |
| Basal metabolic rate (BMR) (Kcal/day) | 1391.00 [1357.00, 1989.50] | 1664.50 [1579.75, 1801.25] | 0.495 |
| Basal metabolic rate – RER | 0.88 [0.87, 0.89] | 0.87 [0.86, 0.89] | 0.558 |
| RER <0.65 (min) | 44.00 [4.50, 63.50] | 16.50 [4.25, 42.00] | 0.845 |
| 24Hour EE (Non Exercise) (Kcal/day) | 1481.00 [1435.00, 1640.00] | 1968.00 [1766.00, 2083.00] | 0.086 |
| 24Hour EE (Non Exercise) by kg of FFM (Kcal/kg/day) | 38.84 [36.53, 39.62] | 39.81 [38.86, 42.82] | 0.54 |
| SMR (Kcal/day) | 1162.00 [1141.00, 1255.00] | 1501.00 [1347.50, 1720.50] | 0.142 |
| SMR_by kg of FFM (Kcal/kg_FFM_/day) | 29.24 [29.04, 31.83] | 31.51 [29.17, 32.21] | 0.624 |
| SMR adjusted by body composition | 1175.27 [1086.48, 1183.88] | 1419.54 [1319.37, 1539.65] | 0.037 |
| BMR (Kcal/day) | 1363.00 [1351.00, 1391.00] | 1664.50 [1579.75, 1801.25] | 0.111 |
| BMR/kg of FFM (Kcal/kg_FFM_/day) | 36.25 [34.87, 36.46] | 35.25 [34.02, 38.36] | 0.624 |
| BMR adjusted by body composition | 1363.73 [1252.58, 1367.76] | 1583.88 [1471.40, 1771.98] | 0.142 |
| RMR (Kcal/day) | 1502.00 [1259.00, 1516.00] | 1609.00 [1528.25, 1851.25] | 0.178 |
| RMR/kg of FFM (Kcal/kg_FFM_/day) | 34.63 [34.29, 40.56] | 35.52 [33.22, 36.50] | 0.713 |
| RMR adjusted by body composition | 1347.72 [1343.84, 1383.44] | 1551.33 [1446.35, 1759.33] | 0.142 |
| Energy Balance (Kcal/day) | 35.00 [-53.00, 62.00] | 39.00 [-7.00, 60.75] | 0.922 |
| Pancreas volume (cm^3) | 78484.65 [63191.89, 94700.95] | 43322.21 [35921.24, 48231.46] | 0.001 |
| Liver volume (cm^3) | 1185595.77 [1128007.52, 1661495.92] | 1437953.81 [1290598.69, 1725460.91] | 0.329 |
| Right Kidney volume (cm^3) | 140010.99 [118857.52, 166333.67] | 159300.90 [146297.12, 177952.06] | 0.143 |
| Left Kidney volume (cm^3) | 122221.55 [118685.23, 173808.59] | 178556.03 [159871.62, 204226.50] | 0.064 |
| Spleen volume (cm^3) | 192669.65 [119813.74, 265899.14] | 263546.21 [230444.58, 320500.69] | 0.143 |
| Pancreas/Body Weight (cm^3/kg) | 1100.96 [968.62, 1312.48] | 563.33 [490.38, 656.39] | 0.001 |
| Liver/ Body Weight (cm^3/kg) | 19815.41 [18790.20, 21159.16] | 21002.08 [19064.95, 21357.71] | 0.558 |
| Right_Kidney/ Body Weight (cm^3/kg) | 2136.52 [1767.30, 2269.87] | 2273.41 [1965.35, 2593.80] | 0.172 |
| left_Kidney/ Body Weight (cm^3/kg) | 2078.60 [1740.36, 2435.65] | 2486.42 [2286.47, 2904.74] | 0.118 |
| Spleen/ Body Weight (cm^3/kg) | 3004.76 [2203.73, 3138.55] | 3592.92 [3196.23, 4247.35] | 0.051 |
| Pancreas/BMI (cm^3/kg/m^2) | 3076.62 [2481.98, 3488.63] | 1525.48 [1339.41, 1758.25] | 0.001 |
| Liver/BMI (cm^3/kg/m^2) | 54809.03 [51797.34, 56328.83] | 55470.25 [52037.42, 57176.10] | 0.77 |
| Right_Kidney/BMI (cm^3/kg/m^2) | 5094.78 [4954.69, 5442.24] | 6507.34 [5584.85, 7081.84] | 0.051 |
| left_Kidney/BMI (cm^3/kg/m^2) | 5265.60 [4941.83, 5946.78] | 7256.31 [5875.16, 8415.28] | 0.051 |
| Spleen/BMI (cm^3/kg/m^2) | 8700.83 [5487.32, 9382.05] | 9991.14 [8330.76, 11798.25] | 0.051 |
| Pancreas/FFM (cm^3/g) | 1.74 [1.60, 1.95] | 0.85 [0.80, 1.09] | 0.001 |
| Liver/FFM (cm^3/g) | 32.42 [30.45, 34.64] | 31.18 [29.15, 36.46] | 0.922 |
| Right_Kidney/FFM (cm^3/g) | 2.97 [2.91, 3.52] | 3.48 [3.30, 4.19] | 0.097 |
| Left_Kidney/FFM (cm^3/g) | 3.34 [3.07, 3.49] | 4.09 [3.64, 4.42] | 0.019 |
| Spleen/FFM (cm^3/g) | 4.21 [3.44, 5.64] | 5.81 [4.91, 6.84] | 0.04 |
| Muscle_sum_Area/FFM (cm^2/g) | 0.63 [0.58, 0.67] | 0.59 [0.49, 0.63] | 0.143 |
| Muscle_sum_Volume/FFM (cm^3/g) | 51.12 [47.19, 54.26] | 47.59 [39.80, 50.82] | 0.118 |
| Muscle_sum_Area/BW (cm^2/g) | 387.34 [347.93, 451.74] | 347.81 [302.04, 395.33] | 0.329 |
| Muscle_sum_Volume/BW (cm^3/g) | 31008.49 [28205.28, 36437.64] | 28218.30 [24583.71, 31811.60] | 0.329 |
| CGM_glu_mean_all (mg/dL) | 84.93 [81.07, 90.37] | 195.90 [164.97, 211.26] | 0.002 |
| CGM_glu_SD_all (mg/dL) | 15.38 [14.26, 19.26] | 74.05 [69.83, 82.32] | 0.002 |
| CGM_glu_n_all | 499.00 [482.50, 505.50] | 2462.00 [2162.50, 2688.50] | 0.002 |
| CGM_glu_CV_all (mg/dL) | 19.09 [17.14, 22.33] | 38.45 [36.56, 45.00] | 0.002 |
| CGM_NotChamber_%181-250_(%) | 0.00 [0.00, 0.00] | 27.18 [22.60, 29.69] | 0.001 |
| CGM_NotChamber_%54-69_(%) | 11.74 [9.86, 21.19] | 2.82 [1.19, 6.62] | 0.032 |
| CGM_NotChamber_%70-180_(%) | 78.61 [70.21, 88.54] | 43.34 [38.22, 54.35] | 0.004 |
| CGM_NotChamber_%<54_(%) | 1.60 [0.90, 2.55] | 1.14 [0.73, 3.21] | 0.83 |
| CGM_NotChamber_%>250_(%) | 0.00 [0.00, 0.00] | 23.16 [13.36, 29.55] | 0.001 |
| eGDR (mg/kg/min) | 9.87 [8.90, 10.28] | 7.88 [6.07, 8.99] | 0.032 |
| eIS-nf | 2.73 [2.46, 3.00] | 1.21 [0.93, 1.52] | 0.001 |
| eIS_noA | 2.89 [2.56, 3.13] | 1.21 [0.93, 1.52] | 0.001 |
| Estimated Blood glucose from HbA1c (mg/dL) | 99.67 [95.37, 105.41] | 185.77 [152.76, 227.39] | 0.001 |
| Average BG in chamber (mg/dL) | 85.93 [80.32, 89.74] | 186.14 [171.89, 211.77] | 0.006 |
| EV concentration (x10^11^ particles/mL) | 1.25 [1.03, 1.60] | 0.82 [0.53, 1.61] | 0.205 |
| EV size – mean (nm) | 212.10 [185.85, 238.20] | 201.95 [192.50, 217.22] | 0.696 |
| EV size – mode (nm) | 145.00 [139.00, 150.35] | 143.80 [140.80, 155.38] | 0.807 |

Each variable is expressed as a median and interquartile range [IQR]. Significance (p-values) are obtained by applying a Mann-Whitney U Test.

Abbreviations: Avg: average, bpm: beats per minute, BMI: body mass index, BMR: basal metabolic rate, CGM: continuous glucose monitoring, DEXA: dual-energy x-ray absorptiometry, EE: energy expenditure, eIS-nf: estimated insulin sensitivity index with variables not requiring fasting state, eIS-noA: estimated insulin sensitivity index not including adiponectin, HbA1c: glycated hemoglobin, IMAT: intermuscular adipose tissue, MRE: magnetic resonance elastography, RER: respiratory exchange ratio, RMR: resting metabolic rate, SMR: sleeping metabolic rate, SAT: subcutaneous adipose tissue, VAT: visceral adipose tissue
