# Supplementary material for: The proteome and phosphoproteome of circulating extracellular vesicle-enriched preparations are associated with characteristic clinical features in type 1 diabetes
Source: Front Endocrinol (Lausanne). 2023 Jul 28;14:1219293. doi: 10.3389/fendo.2023.1219293 (PMC10417723; doi:10.3389/fendo.2023.1219293)
Supplement: Supplementary file 3 [file Table_2.docx]

**Supplementary Table ST2**: Differentially abundant proteins in circulating EV-enriched preparations (T1D versus Controls)

| **Uniprot ID** | **Gene Symbol** | **Log2FC** | **log2 Ave Expr** | **P.Value** | **FDR** |
| --- | --- | --- | --- | --- | --- |
| P30101 | PDIA3 | 2.87 | 0.50 | 6.75E-10 | 6.53E-07 |
| P61158 | ACTR3 | 2.78 | 0.25 | 1.96E-09 | 8.23E-07 |
| P02745 | C1QA | 2.73 | -0.03 | 7.70E-09 | 2.19E-06 |
| P28838 | LAP3 | 2.72 | 0.13 | 1.24E-09 | 6.53E-07 |
| P00747 | PLG | 2.48 | 0.32 | 5.86E-09 | 2.05E-06 |
| O60229 | KALRN | 2.39 | -0.51 | 1.55E-06 | 0.00022 |
| P62834 | RAP1A | 2.38 | -0.05 | 2.72E-07 | 5.20E-05 |
| P05141 | SLC25A5 | 2.36 | -0.18 | 1.80E-06 | 0.00023 |
| Q9BS26 | ERP44 | 2.34 | 0.66 | 1.18E-08 | 2.75E-06 |
| Q8N1N4 | KRT78 | 2.34 | 0.83 | 5.49E-10 | 6.53E-07 |
| A0A0B4J1Y8 | IGLV9-49 | 2.23 | -0.19 | 1.12E-06 | 0.00017 |
| O14950 | MYL12A | 2.18 | 0.20 | 4.06E-05 | 0.00246 |
| P30048 | PRDX3 | 2.16 | -0.04 | 5.42E-04 | 0.01866 |
| O75223 | GGCT | 2.13 | 0.37 | 1.26E-05 | 0.00115 |
| Q06187 | BTK | 2.13 | 0.49 | 1.08E-06 | 0.00017 |
| Q05193 | DNM1 | 2.09 | -0.31 | 4.09E-04 | 0.01505 |
| P51790 | CLCN3 | 2.06 | -0.13 | 2.19E-03 | 0.04778 |
| Q9Y210 | TRPC6 | 2.05 | -0.08 | 2.31E-03 | 0.04900 |
| P31944 | CASP14 | 2.02 | 0.43 | 4.29E-05 | 0.00250 |
| P39060 | COL18A1 | 2.01 | -0.19 | 3.28E-05 | 0.00222 |
| Q13576 | IQGAP2 | 1.98 | -0.81 | 5.52E-08 | 0.00001 |
| P30042 | GATD3 | 1.96 | 0.11 | 1.99E-03 | 0.04492 |
| O43639 | NCK2 | 1.94 | 0.57 | 1.18E-04 | 0.00588 |
| P61163 | ACTR1A | 1.94 | 0.58 | 3.59E-05 | 0.00228 |
| P16284 | PECAM1 | 1.93 | -0.17 | 1.63E-05 | 0.00142 |
| P00387 | CYB5R3 | 1.89 | -0.81 | 3.12E-05 | 0.00218 |
| O60664 | PLIN3 | 1.88 | 0.78 | 2.06E-05 | 0.00173 |
| P61088 | UBE2N | 1.87 | -0.36 | 1.77E-03 | 0.04323 |
| Q9ULI3 | HEG1 | 1.87 | 0.61 | 2.23E-04 | 0.00916 |
| Q4KMP7 | TBC1D10B | 1.87 | 0.63 | 6.29E-05 | 0.00338 |
| Q9Y5W7 | SNX14 | 1.86 | -0.23 | 7.61E-03 | 0.09559 |
| P20936 | RASA1 | 1.86 | 0.67 | 2.54E-05 | 0.00204 |
| P60520 | GABARAPL2 | 1.85 | -0.16 | 2.98E-04 | 0.01139 |
| Q01518 | CAP1 | 1.80 | 0.50 | 1.15E-03 | 0.03123 |
| Q9Y376 | CAB39 | 1.79 | 0.04 | 9.27E-04 | 0.02734 |
| Q9NS84 | CHST7 | 1.75 | 0.32 | 2.49E-04 | 0.01007 |
| P35613 | BSG | 1.74 | 0.51 | 1.37E-03 | 0.03544 |
| P13671 | C6 | 1.72 | -0.50 | 5.91E-05 | 0.00326 |
| P55072 | VCP | 1.71 | -0.14 | 8.58E-04 | 0.02649 |
| Q14141 | SEPTIN6 | 1.70 | 0.75 | 9.50E-04 | 0.02734 |
| P08779 | KRT16 | 1.69 | 0.27 | 2.82E-05 | 0.00204 |
| P21333 | FLNA | 1.69 | -0.39 | 1.74E-03 | 0.04298 |
| P06576 | ATP5F1B | 1.68 | -0.76 | 2.72E-05 | 0.00204 |
| P60981 | DSTN | 1.66 | -0.15 | 3.98E-03 | 0.06558 |
| P30613 | PKLR | 1.65 | -0.54 | 6.79E-05 | 0.00356 |
| Q13637 | RAB32 | 1.64 | -0.83 | 5.19E-05 | 0.00295 |
| P61106 | RAB14 | 1.63 | -0.78 | 6.37E-04 | 0.02114 |
| P23490 | LORICRIN | 1.62 | 0.07 | 9.51E-04 | 0.02734 |
| Q13126 | MTAP | 1.61 | -0.66 | 7.96E-04 | 0.02495 |
| Q13387 | MAPK8IP2 | 1.61 | 0.47 | 2.47E-03 | 0.04978 |
| Q9NV96 | TMEM30A | 1.58 | 0.28 | 3.40E-03 | 0.05886 |
| P07948 | LYN | 1.58 | 0.63 | 2.15E-04 | 0.00902 |
| Q9H479 | FN3K | 1.54 | -0.41 | 5.45E-03 | 0.07948 |
| P22748 | CA4 | 1.53 | 0.55 | 2.56E-04 | 0.01015 |
| P49006 | MARCKSL1 | 1.52 | -0.17 | 7.88E-03 | 0.09677 |
| Q92882 | OSTF1 | 1.52 | -0.17 | 5.22E-03 | 0.07769 |
| Q8NF50 | DOCK8 | 1.51 | -0.90 | 6.76E-04 | 0.02183 |
| P49247 | RPIA | 1.50 | -0.16 | 9.31E-04 | 0.02734 |
| P31939 | ATIC | 1.50 | -0.04 | 2.09E-04 | 0.00902 |
| Q08495 | DMTN | 1.50 | -0.05 | 6.26E-03 | 0.08496 |
| Q9H7D0 | DOCK5 | 1.49 | 0.48 | 1.70E-04 | 0.00778 |
| Q15691 | MAPRE1 | 1.49 | 0.27 | 7.51E-04 | 0.02389 |
| P61604 | HSPE1 | 1.48 | 0.63 | 4.00E-03 | 0.06558 |
| P14868 | DARS1 | 1.48 | 0.45 | 1.85E-06 | 0.00023 |
| P04899 | GNAI2 | 1.47 | -0.09 | 2.25E-03 | 0.04869 |
| P06241 | FYN | 1.46 | 0.48 | 7.99E-03 | 0.09747 |
| P22694 | PRKACB | 1.45 | 0.11 | 7.01E-03 | 0.09079 |
| P55209 | NAP1L1 | 1.44 | -0.51 | 2.99E-03 | 0.05509 |
| P15907 | ST6GAL1 | 1.43 | 0.48 | 5.59E-03 | 0.07960 |
| P12821 | ACE | 1.41 | -0.30 | 3.15E-04 | 0.01181 |
| P06702 | S100A9 | 1.41 | 0.10 | 9.43E-04 | 0.02734 |
| O60427 | FADS1 | 1.41 | -0.43 | 2.40E-03 | 0.04905 |
| P04792 | HSPB1 | 1.40 | 0.41 | 2.75E-04 | 0.01068 |
| O15162 | PLSCR1 | 1.40 | -0.59 | 4.10E-05 | 0.00246 |
| P40189 | IL6ST | 1.40 | -0.32 | 4.65E-04 | 0.01654 |
| P25942 | CD40 | 1.38 | -0.63 | 2.30E-03 | 0.04900 |
| P21281 | ATP6V1B2 | 1.37 | 0.21 | 9.11E-06 | 0.00091 |
| P07197 | NEFM | 1.37 | 0.07 | 7.35E-03 | 0.09373 |
| P13804 | ETFA | 1.36 | 0.89 | 1.59E-04 | 0.00742 |
| P18206 | VCL | 1.35 | -0.63 | 8.19E-03 | 0.09772 |
| P48163 | ME1 | 1.35 | 0.34 | 1.61E-03 | 0.04122 |
| P0DOX2 | NA | 1.33 | -0.62 | 1.21E-03 | 0.03266 |
| P24557 | TBXAS1 | 1.32 | -0.10 | 5.20E-03 | 0.07769 |
| Q13094 | LCP2 | 1.32 | 0.98 | 1.56E-04 | 0.00742 |
| P17900 | GM2A | 1.32 | 0.49 | 2.33E-03 | 0.04900 |
| Q9BT78 | COPS4 | 1.32 | -0.99 | 1.94E-03 | 0.04438 |
| O75964 | ATP5MG | 1.31 | 0.43 | 5.29E-03 | 0.07769 |
| P08962 | CD63 | 1.29 | 0.45 | 3.82E-03 | 0.06414 |
| O75116 | ROCK2 | 1.29 | -0.25 | 6.34E-03 | 0.08496 |
| P34910 | EVI2B | 1.29 | -0.53 | 2.80E-03 | 0.05315 |
| O75460 | ERN1 | 1.28 | -0.86 | 1.93E-03 | 0.04438 |
| Q9H1B5 | XYLT2 | 1.27 | -0.36 | 2.12E-03 | 0.04694 |
| P17980 | PSMC3 | 1.27 | -0.64 | 8.33E-03 | 0.09829 |
| Q15208 | STK38 | 1.27 | 0.45 | 8.29E-03 | 0.09829 |
| P43304 | GPD2 | 1.27 | 0.84 | 1.70E-03 | 0.04260 |
| Q9H0U4 | RAB1B | 1.25 | -0.33 | 8.47E-03 | 0.09870 |
| P16402 | H1-3 | 1.24 | -0.18 | 1.32E-03 | 0.03471 |
| Q9Y4G6 | TLN2 | 1.23 | -0.58 | 5.15E-03 | 0.07769 |
| P14550 | AKR1A1 | 1.22 | 0.54 | 6.40E-03 | 0.08496 |
| P35241 | RDX | 1.22 | -0.37 | 8.51E-03 | 0.09870 |
| Q14697 | GANAB | 1.22 | -0.41 | 2.78E-03 | 0.05315 |
| P40926 | MDH2 | 1.21 | -0.56 | 2.11E-04 | 0.00902 |
| P06753 | TPM3 | 1.21 | -0.56 | 7.44E-03 | 0.09408 |
| P37802 | TAGLN2 | 1.21 | 0.28 | 8.19E-03 | 0.09772 |
| Q99623 | PHB2 | 1.21 | 0.88 | 5.58E-03 | 0.07960 |
| Q969T9 | WBP2 | 1.20 | -0.96 | 1.80E-03 | 0.04350 |
| P35611 | ADD1 | 1.18 | -0.57 | 3.31E-03 | 0.05877 |
| P02774 | GC | 1.16 | -1.02 | 4.95E-04 | 0.01731 |
| Q6YHK3 | CD109 | 1.16 | -0.80 | 7.36E-03 | 0.09373 |
| P15311 | EZR | 1.15 | 0.48 | 5.65E-03 | 0.07960 |
| P06396 | GSN | 1.15 | -0.50 | 6.38E-03 | 0.08496 |
| P35749 | MYH11 | 1.14 | 0.91 | 3.94E-03 | 0.06558 |
| P05164 | MPO | 1.14 | -0.66 | 6.19E-03 | 0.08496 |
| P16671 | CD36 | 1.12 | -0.81 | 4.60E-03 | 0.07266 |
| Q8IXY8 | PPIL6 | 1.12 | -0.61 | 6.93E-03 | 0.09034 |
| Q07960 | ARHGAP1 | 1.11 | -1.09 | 3.23E-03 | 0.05841 |
| O75390 | CS | 1.10 | -0.48 | 4.15E-03 | 0.06643 |
| P02788 | LTF | 1.10 | -0.57 | 2.58E-03 | 0.05100 |
| O43681 | GET3 | 1.10 | 0.26 | 1.01E-03 | 0.02829 |
| P25815 | S100P | 1.05 | -0.62 | 5.62E-03 | 0.07960 |
| Q8WXH0 | SYNE2 | 1.05 | -0.98 | 2.67E-03 | 0.05181 |
| P50990 | CCT8 | 1.04 | -0.10 | 3.33E-03 | 0.05877 |
| C9J442 | IgGalpha2 | 1.03 | -0.18 | 6.18E-03 | 0.08496 |
| Q9NYC9 | DNAH9 | 1.03 | -1.04 | 6.59E-03 | 0.08685 |
| Q2M2E5 | NA | 1.03 | -0.76 | 8.17E-03 | 0.09772 |
| P08493 | MGP | 1.02 | -0.20 | 4.10E-03 | 0.06629 |
| P13473 | LAMP2 | 1.00 | -0.85 | 5.61E-03 | 0.07960 |
| P02750 | LRG1 | 0.97 | -0.82 | 7.77E-03 | 0.09628 |
| P17213 | BPI | 0.96 | -0.40 | 7.37E-03 | 0.09373 |
| Q96PD5 | PGLYRP2 | 0.94 | -0.76 | 4.89E-03 | 0.07599 |
| P54709 | ATP1B3 | 0.92 | 0.91 | 3.61E-03 | 0.06154 |
| O14818 | PSMA7 | 0.88 | -1.01 | 8.18E-03 | 0.09772 |
| Q9Y277 | VDAC3 | 0.87 | -0.31 | 3.65E-03 | 0.06174 |
| O43399 | TPD52L2 | 0.80 | -0.01 | 5.22E-03 | 0.07769 |
| Q9HD89 | RETN | 0.75 | 0.81 | 8.51E-03 | 0.09870 |
| Q9NSD9 | FARSB | -0.71 | -0.88 | 5.11E-03 | 0.07769 |
| Q9P2S5 | WRAP73 | -0.77 | -0.17 | 1.88E-03 | 0.04411 |
| Q9BX46 | RBM24 | -0.97 | -0.25 | 2.84E-03 | 0.05315 |
| O43424 | GRID2 | -1.00 | -0.82 | 2.41E-03 | 0.04905 |
| Q6UWP8 | SBSN | -1.00 | -0.94 | 5.29E-03 | 0.07769 |
| Q96IR7 | HPDL | -1.01 | -0.72 | 4.59E-04 | 0.01654 |
| P09960 | LTA4H | -1.02 | -0.43 | 2.56E-03 | 0.05100 |
| P54707 | ATP12A | -1.03 | -0.47 | 6.62E-03 | 0.08685 |
| P25789 | PSMA4 | -1.04 | -1.11 | 2.82E-03 | 0.05315 |
| O15067 | PFAS | -1.05 | -0.99 | 6.36E-03 | 0.08496 |
| P17010 | ZFX | -1.08 | -0.80 | 9.76E-04 | 0.02768 |
| Q99426 | TBCB | -1.09 | -0.15 | 6.45E-04 | 0.02114 |
| P21854 | CD72 | -1.09 | -0.86 | 6.18E-03 | 0.08496 |
| Q99459 | CDC5L | -1.12 | -0.80 | 1.25E-03 | 0.03316 |
| P08574 | CYC1 | -1.15 | -1.07 | 4.49E-03 | 0.07147 |
| Q8IU68 | TMC8 | -1.17 | -0.89 | 6.34E-03 | 0.08496 |
| Q5VTH9 | DNAI4 | -1.17 | -1.04 | 4.11E-03 | 0.06629 |
| Q86W11 | ZSCAN30 | -1.17 | -0.71 | 3.38E-03 | 0.05886 |
| P08397 | HMBS | -1.19 | -0.87 | 5.21E-03 | 0.07769 |
| Q9HBI6 | CYP4F11 | -1.23 | -0.77 | 3.42E-03 | 0.05886 |
| P50416 | CPT1A | -1.25 | -0.83 | 2.01E-04 | 0.00898 |
| P02743 | APCS | -1.26 | -0.86 | 2.91E-03 | 0.05401 |
| Q92835 | INPP5D | -1.41 | -1.09 | 1.12E-05 | 0.00107 |
| Q13200 | PSMD2 | -1.44 | -0.51 | 4.80E-03 | 0.07520 |
| Q5T5U3 | ARHGAP21 | -1.44 | 0.00 | 2.60E-03 | 0.05102 |
| P12109 | COL6A1 | -1.46 | 0.50 | 7.78E-03 | 0.09628 |
| P04085 | PDGFA | -1.46 | -0.84 | 1.23E-04 | 0.00601 |
| Q96J01 | THOC3 | -1.48 | -0.51 | 1.07E-03 | 0.02950 |
| P42574 | CASP3 | -1.49 | 0.67 | 2.12E-03 | 0.04694 |
| Q9BY15 | ADGRE3 | -1.50 | 0.22 | 7.80E-03 | 0.09628 |
| Q02818 | NUCB1 | -1.54 | -1.02 | 3.93E-07 | 0.00007 |
| P01920 | HLA-DQB1 | -1.58 | 0.43 | 1.89E-03 | 0.04411 |
| P36969 | GPX4 | -1.59 | 0.26 | 3.02E-03 | 0.05517 |
| O00241 | SIRPB1 | -1.62 | -0.72 | 7.94E-06 | 0.00083 |
| O75122 | CLASP2 | -1.64 | 0.03 | 3.30E-03 | 0.05877 |
| P42858 | HTT | -1.64 | -0.86 | 1.64E-03 | 0.04159 |
| P80108 | GPLD1 | -1.68 | 0.35 | 2.62E-05 | 0.00204 |
| P04233 | CD74 | -1.78 | 0.09 | 2.37E-03 | 0.04905 |
| Q16531 | DDB1 | -1.80 | -0.84 | 5.59E-04 | 0.01892 |
| P04229 | HLA-DRB1 | -1.86 | 0.02 | 1.89E-03 | 0.04411 |
| Q99969 | RARRES2 | -1.95 | -0.42 | 4.67E-06 | 0.00054 |
| Q9Y6Z7 | COLEC10 | -1.96 | 0.59 | 1.12E-04 | 0.00573 |
| Q86VD1 | MORC1 | -2.22 | 0.23 | 3.58E-05 | 0.00228 |
| P07954 | FH | -2.63 | -0.26 | 6.79E-06 | 0.00075 |
| O75533 | SF3B1 | -2.64 | 0.52 | 1.01E-09 | 6.53E-07 |
| P02794 | FTH1 | -2.80 | -0.21 | 8.33E-09 | 2.19E-06 |

Abbreviations: Log2FC: base 2 logarithm of fold change, Log2 Ave Expr: base 2 logarithm of average expression, FDR: False discovery rate.
