# Supplementary material for: The proteome and phosphoproteome of circulating extracellular vesicle-enriched preparations are associated with characteristic clinical features in type 1 diabetes
Source: Front Endocrinol (Lausanne). 2023 Jul 28;14:1219293. doi: 10.3389/fendo.2023.1219293 (PMC10417723; doi:10.3389/fendo.2023.1219293)
Supplement: Supplementary file 6 [file Table_5.docx]

**Supplementary Table 5**

List of 41 proteins selected by connectivity and gene significance associated with % of time >250 mg/dl in the Tan module.

| Module | UniprotID | GeneSymbol | ProteinName | kIN | GS.TargetTrait | p.GS.TargetTrait |
| --- | --- | --- | --- | --- | --- | --- |
| tan | Q9NV96 | TMEM30A | transmembrane protein 30A | 5.285848038 | 0.580760272 | 0.01832512 |
| tan | Q9NS84 | CHST7 | carbohydrate sulfotransferase 7 | 6.94834112 | 0.748522134 | 0.000850622 |
| tan | Q9H7D0.1 | DOCK5 | dedicator of cytokinesis 5 | 8.141138234 | 0.742887762 | 0.000977134 |
| tan | Q9BS26 | ERP44 | endoplasmic reticulum protein 44 | 15.57137789 | 0.942701309 | 4.67E-08 |
| tan | Q8NF50.1 | DOCK8 | dedicator of cytokinesis 8 | 4.478433232 | 0.759313251 | 0.000645708 |
| tan | Q8N1N4 | KRT78 | keratin 78 | 13.89340379 | 0.978705063 | 5.03E-11 |
| tan | Q15691 | MAPRE1 | microtubule associated protein RP/EB family member 1 | 10.22166709 | 0.635453911 | 0.008160998 |
| tan | Q14141.2 | SEPTIN6 | septin 6 | 7.372036228 | 0.673692834 | 0.004220007 |
| tan | Q13576.1 | IQGAP2 | IQ motif containing GTPase activating protein 2 | 14.70285046 | 0.935076185 | 0.00000011 |
| tan | Q13094 | LCP2 | lymphocyte cytosolic protein 2 | 11.58505379 | 0.744273185 | 0.000944684 |
| tan | Q06187 | BTK | Bruton tyrosine kinase | 10.93621463 | 0.829939053 | 0.0000695 |
| tan | P62834 | RAP1A | RAP1A, member of RAS oncogene family | 6.681169258 | 0.829596816 | 0.0000704 |
| tan | P61163 | ACTR1A | actin related protein 1A | 13.44570559 | 0.811652089 | 0.000134843 |
| tan | P61158 | ACTR3 | actin related protein 3 | 15.80326339 | 0.935675011 | 0.000000103 |
| tan | P50990 | CCT8 | chaperonin containing TCP1 subunit 8 | 10.53356037 | 0.6064271 | 0.012757939 |
| tan | P50395.1 | GDI2 | GDP dissociation inhibitor 2 | 7.337386066 | 0.575162653 | 0.019757277 |
| tan | P31944 | CASP14 | caspase 14 | 6.061328729 | 0.734815456 | 0.001184862 |
| tan | P30101 | PDIA3 | protein disulfide isomerase family A member 3 | 14.21009437 | 0.959765219 | 4.11E-09 |
| tan | P28838 | LAP3 | leucine aminopeptidase 3 | 15.06617901 | 0.958003456 | 5.53E-09 |
| tan | P22748 | CA4 | carbonic anhydrase 4 | 5.85513437 | 0.754031737 | 0.000740218 |
| tan | P21281 | ATP6V1B2 | ATPase H+ transporting V1 subunit B2 | 12.30109339 | 0.886403238 | 0.00000482 |
| tan | P20936.1 | RASA1 | RAS p21 protein activator 1 | 11.38173198 | 0.80615135 | 0.000162388 |
| tan | P16284 | PECAM1 | platelet and endothelial cell adhesion molecule 1 | 13.41528703 | 0.797703769 | 0.000213685 |
| tan | P14868 | DARS1 | aspartyl-tRNA synthetase 1 | 14.76473532 | 0.901435751 | 0.00000186 |
| tan | P13804.1 | ETFA | electron transfer flavoprotein subunit alpha | 7.178251305 | 0.797433343 | 0.000215527 |
| tan | P13671 | C6 | complement C6 | 5.311049628 | 0.821333669 | 0.0000958 |
| tan | P12821.1 | ACE | angiotensin I converting enzyme | 8.848270098 | 0.72878539 | 0.001362482 |
| tan | P08779 | KRT16 | keratin 16 | 9.55245266 | 0.839041335 | 0.0000485 |
| tan | P07948.2 | LYN | LYN proto-oncogene, Src family tyrosine kinase | 12.13110112 | 0.71312559 | 0.001927144 |
| tan | P06576 | ATP5F1B | ATP synthase F1 subunit beta | 10.0740158 | 0.746516352 | 0.000894019 |
| tan | P05141 | SLC25A5 | solute carrier family 25 member 5 | 14.42456885 | 0.842011795 | 0.0000429 |
| tan | P04899.1 | GNAI2 | G protein subunit alpha i2 | 8.55187771 | 0.584226242 | 0.01747959 |
| tan | P04792 | HSPB1 | heat shock protein family B (small) member 1 | 8.9726762 | 0.72396513 | 0.001519583 |
| tan | P02774.3 | GC | GC vitamin D binding protein | 4.511869587 | 0.769082132 | 0.000497004 |
| tan | P02745 | C1QA | complement C1q A chain | 16.40919343 | 0.935265781 | 0.000000108 |
| tan | P00387.3 | CYB5R3 | cytochrome b5 reductase 3 | 10.0471581 | 0.789587621 | 0.000274966 |
| tan | O75223.1 | GGCT | gamma-glutamylcyclotransferase | 8.550440969 | 0.798118534 | 0.000210887 |
| tan | O15162 | PLSCR1 | phospholipid scramblase 1 | 5.332588633 | 0.814763879 | 0.000121064 |
| tan | O14950 | MYL12A | myosin light chain 12A | 8.623642782 | 0.79565087 | 0.000227994 |
| tan | O14818.1 | PSMA7 | proteasome 20S subunit alpha 7 | 7.603398811 | 0.592073895 | 0.015677129 |
| tan | A0A0B4J1Y8 | NA | NA | 10.43161579 | 0.88717455 | 0.00000461 |

Abbreviations: **kIN**: the connectivity of the nodes (protein here), that is the sum of the adjacency to the other nodes. **GS**: Gene significance -- the correlation between the protein and the clinical trait. **p.GS**: the p-value for null hypothesis testing of the correlation between the protein and the target clinical trait.

**Supplementary Table 6**

List of 36 proteins selected by connectivity and gene significance associated with Pancreas size in the Tan module.

| Module | UniprotID | GeneSymbol | ProteinName | kIN | GS.TargetTrait | p.GS.TargetTrait |
| --- | --- | --- | --- | --- | --- | --- |
| tan | Q9NV96 | TMEM30A | transmembrane protein 30A | 5.285848038 | -0.501696335 | 0.047700891 |
| tan | Q9NS84 | CHST7 | carbohydrate sulfotransferase 7 | 6.94834112 | -0.657893148 | 0.005601665 |
| tan | Q9H7D0.1 | DOCK5 | dedicator of cytokinesis 5 | 8.141138234 | -0.611713388 | 0.011797442 |
| tan | Q9BS26 | ERP44 | endoplasmic reticulum protein 44 | 15.57137789 | -0.72988224 | 0.001328653 |
| tan | Q8NF50.1 | DOCK8 | dedicator of cytokinesis 8 | 4.478433232 | -0.5900411 | 0.016129447 |
| tan | Q8N1N4 | KRT78 | keratin 78 | 13.89340379 | -0.800576497 | 0.000194919 |
| tan | Q15691 | MAPRE1 | microtubule associated protein RP/EB family member 1 | 10.22166709 | -0.502424556 | 0.047327036 |
| tan | Q14141.2 | SEPTIN6 | septin 6 | 7.372036228 | -0.510185906 | 0.043477 |
| tan | Q13576.1 | IQGAP2 | IQ motif containing GTPase activating protein 2 | 14.70285046 | -0.76830842 | 0.000507642 |
| tan | Q13094 | LCP2 | lymphocyte cytosolic protein 2 | 11.58505379 | -0.568813132 | 0.021485016 |
| tan | Q06187 | BTK | Bruton tyrosine kinase | 10.93621463 | -0.749620211 | 0.000827605 |
| tan | P62834 | RAP1A | RAP1A, member of RAS oncogene family | 6.681169258 | -0.858255022 | 0.000021 |
| tan | P61163 | ACTR1A | actin related protein 1A | 13.44570559 | -0.558910339 | 0.02440959 |
| tan | P61158 | ACTR3 | actin related protein 3 | 15.80326339 | -0.773590286 | 0.000438607 |
| tan | P31944 | CASP14 | caspase 14 | 6.061328729 | -0.621644246 | 0.010147233 |
| tan | P30101 | PDIA3 | protein disulfide isomerase family A member 3 | 14.21009437 | -0.763610824 | 0.000576335 |
| tan | P28838 | LAP3 | leucine aminopeptidase 3 | 15.06617901 | -0.758117657 | 0.000666181 |
| tan | P21281 | ATP6V1B2 | ATPase H+ transporting V1 subunit B2 | 12.30109339 | -0.693008812 | 0.002917236 |
| tan | P20936.1 | RASA1 | RAS p21 protein activator 1 | 11.38173198 | -0.584150658 | 0.0174977 |
| tan | P16284 | PECAM1 | platelet and endothelial cell adhesion molecule 1 | 13.41528703 | -0.635524896 | 0.008151653 |
| tan | P14868 | DARS1 | aspartyl-tRNA synthetase 1 | 14.76473532 | -0.770354855 | 0.000479905 |
| tan | P13804.1 | ETFA | electron transfer flavoprotein subunit alpha | 7.178251305 | -0.579599176 | 0.018615351 |
| tan | P13671 | C6 | complement C6 | 5.311049628 | -0.591024403 | 0.0159094 |
| tan | P12821.1 | ACE | angiotensin I converting enzyme | 8.848270098 | -0.529001833 | 0.035119403 |
| tan | P08779 | KRT16 | keratin 16 | 9.55245266 | -0.70237709 | 0.002414807 |
| tan | P07948.2 | LYN | LYN proto-oncogene, Src family tyrosine kinase | 12.13110112 | -0.514365549 | 0.041503488 |
| tan | P06576 | ATP5F1B | ATP synthase F1 subunit beta | 10.0740158 | -0.641122719 | 0.007440996 |
| tan | P05141 | SLC25A5 | solute carrier family 25 member 5 | 14.42456885 | -0.623315243 | 0.009888439 |
| tan | P04792 | HSPB1 | heat shock protein family B (small) member 1 | 8.9726762 | -0.673676941 | 0.004221244 |
| tan | P02774.3 | GC | GC vitamin D binding protein | 4.511869587 | -0.577681428 | 0.019102508 |
| tan | P02745 | C1QA | complement C1q A chain | 16.40919343 | -0.721057152 | 0.001621285 |
| tan | P00387.3 | CYB5R3 | cytochrome b5 reductase 3 | 10.0471581 | -0.564651679 | 0.022679079 |
| tan | O75223.1 | GGCT | gamma-glutamylcyclotransferase | 8.550440969 | -0.710977052 | 0.002017594 |
| tan | O15162 | PLSCR1 | phospholipid scramblase 1 | 5.332588633 | -0.509435813 | 0.043838484 |
| tan | O14950 | MYL12A | myosin light chain 12A | 8.623642782 | -0.581537267 | 0.018132868 |
| tan | A0A0B4J1Y8 | NA | NA | 10.43161579 | -0.714713035 | 0.001862454 |

Abbreviations: **kIN**: the connectivity of the nodes (protein here), that is the sum of the adjacency to the other nodes. **GS**: Gene significance -- the correlation between the protein and the clinical trait. **p.GS**: the p-value for null hypothesis testing of the correlation between the protein and the target clinical trait.

**Supplementary Table 7**

List of 19 proteins selected by connectivity and gene significance associated with Pancreas size in the Salmon color module.

| Module | UniprotID | GeneSymbol | ProteinName | kIN | GS.TargetTrait | p.GS.TargetTrait |
| --- | --- | --- | --- | --- | --- | --- |
| salmon | P04229 | NA | NA | 5.48055322 | 0.828849 | 0.0000724 |
| salmon | Q9Y6N5 | SQOR | sulfide quinone oxidoreductase | 2.848822 | 0.54382706 | 0.02944017 |
| salmon | P36969 | GPX4 | glutathione peroxidase 4 | 5.60684596 | 0.79280478 | 0.00024914 |
| salmon | Q86TC9.1 | MYPN | myopalladin | 0.59506383 | 0.55218091 | 0.02656506 |
| salmon | P02743 | APCS | amyloid P component, serum | 6.49538799 | 0.78741057 | 0.00029367 |
| salmon | Q16531 | DDB1 | damage specific DNA binding protein 1 | 3.74462287 | 0.72248146 | 0.0015708 |
| salmon | Q12846 | STX4 | syntaxin 4 | 2.93084513 | 0.58622365 | 0.01700628 |
| salmon | P01714 | NA | NA | 1.34308174 | 0.68361488 | 0.00350261 |
| salmon | P81605 | DCD | dermcidin | 3.22184274 | 0.53420318 | 0.0330399 |
| salmon | A8K2U0 | A2ML1 | alpha-2-macroglobulin like 1 | 4.20131432 | 0.65864053 | 0.00552908 |
| salmon | P13796 | LCP1 | lymphocyte cytosolic protein 1 | 7.37504805 | 0.65497998 | 0.00589189 |
| salmon | P30486 | NA | NA | 2.67950718 | 0.51540264 | 0.04102439 |
| salmon | P05090 | APOD | apolipoprotein D | 3.16993475 | 0.63523708 | 0.00818959 |
| salmon | P26038 | MSN | moesin | 6.20439533 | 0.5234916 | 0.03742888 |
| salmon | P12724 | RNASE3 | ribonuclease A family member 3 | 2.93354216 | 0.63291445 | 0.00850093 |
| salmon | P12109 | COL6A1 | collagen type VI alpha 1 chain | 3.43052166 | 0.62168898 | 0.01014024 |
| salmon | O75636.1 | FCN3 | ficolin 3 | 4.88287301 | 0.56975113 | 0.0212227 |
| salmon | O15067 | PFAS | phosphoribosylformylglycinamidine synthase | 0.70414458 | 0.5635528 | 0.02300275 |
| salmon | O95445.2 | APOM | apolipoprotein M | 4.33807967 | 0.50906741 | 0.04401684 |

Abbreviations: **kIN**: the connectivity of the nodes (protein here), that is the sum of the adjacency to the other nodes. **GS**: Gene significance -- the correlation between the protein and the clinical trait. **p.GS**: the p-value for null hypothesis testing of the correlation between the protein and the target clinical trait.

**Supplementary Table 8**

List of 25 proteins selected by connectivity and gene significance associated with Pancreas size in the Pink module.

| Module | UniprotID | GeneSymbol | ProteinName | kIN | GS.TargetTrait | p.GS.TargetTrait |
| --- | --- | --- | --- | --- | --- | --- |
| pink | Q9Y6Z7 | COLEC10 | collectin subfamily member 10 | 4.024264 | 0.74085977 | 0.00102627 |
| pink | Q9P2S5 | WRAP73 | WD repeat containing, antisense to TP73 | 5.06762 | 0.63103596 | 0.00875952 |
| pink | Q9NVJ2 | ARL8B | ADP ribosylation factor like GTPase 8B | 0.370222 | 0.58854128 | 0.01646964 |
| pink | Q9NSD9 | FARSB | phenylalanyl-tRNA synthetase subunit beta | 5.556509 | 0.68565278 | 0.00336822 |
| pink | Q99969 | RARRES2 | retinoic acid receptor responder 2 | 5.395851 | 0.77417909 | 0.00043142 |
| pink | Q99459 | CDC5L | cell division cycle 5 like | 6.885067 | 0.66219785 | 0.00519382 |
| pink | Q99426 | TBCB | tubulin folding cofactor B | 1.929007 | 0.6943228 | 0.00284208 |
| pink | Q96IR7 | HPDL | 4-hydroxyphenylpyruvate dioxygenase like | 6.233314 | 0.66270869 | 0.00514705 |
| pink | Q92835 | INPP5D | inositol polyphosphate-5-phosphatase D | 5.412118 | 0.77608387 | 0.00040882 |
| pink | Q86VD1 | MORC1 | MORC family CW-type zinc finger 1 | 6.609833 | 0.58310014 | 0.01775091 |
| pink | Q6UWP8.2 | SBSN | suprabasin | 10.2976 | 0.50906452 | 0.04401824 |
| pink | Q5VTH9.1 | WDR78 | NA | 5.573814 | 0.54978594 | 0.02736633 |
| pink | Q02818 | NUCB1 | nucleobindin 1 | 6.031379 | 0.76808607 | 0.00051073 |
| pink | P80108 | GPLD1 | glycosylphosphatidylinositol specific phospholipase D1 | 2.983188 | 0.74002541 | 0.00104707 |
| pink | P50416 | CPT1A | carnitine palmitoyltransferase 1A | 7.363657 | 0.54738321 | 0.02818864 |
| pink | P32856.3 | STX2 | syntaxin 2 | 2.368885 | 0.64470837 | 0.00701228 |
| pink | P17010 | ZFX | zinc finger protein X-linked | 8.131738 | 0.70751989 | 0.00217035 |
| pink | P08574 | CYC1 | cytochrome c1 | 4.431397 | 0.53167825 | 0.03403736 |
| pink | P07954 | FH | fumarate hydratase | 4.842913 | 0.79996586 | 0.00019879 |
| pink | P04085 | PDGFA | platelet derived growth factor subunit A | 6.732229 | 0.66030441 | 0.00537018 |
| pink | P02794 | FTH1 | ferritin heavy chain 1 | 6.408488 | 0.85456704 | 0.0000249 |
| pink | P01624 | NA | NA | 3.638777 | 0.59614439 | 0.01480115 |
| pink | O94910 | ADGRL1 | adhesion G protein-coupled receptor L1 | 2.305348 | 0.55606189 | 0.02530491 |
| pink | O75533.1 | SF3B1 | splicing factor 3b subunit 1 | 9.265066 | 0.77388504 | 0.00043499 |
| pink | O00241 | SIRPB1 | signal regulatory protein beta 1 | 6.015492 | 0.76935502 | 0.0004933 |

The enrichment analysis with the 25 proteins did not result in significant pathways or biological processes. All 68 proteins did not produce any significant functional categories.

Abbreviations: **kIN**: the connectivity of the nodes (protein here), that is the sum of the adjacency to the other nodes. **GS**: Gene significance -- the correlation between the protein and the clinical trait. **p.GS**: the p-value for null hypothesis testing of the correlation between the protein and the target clinical trait.
